# Administration of adiponectin receptor agonist AdipoRon relieves cancer cachexia by mitigating inflammation in tumour‐bearing mice

**DOI:** 10.1002/jcsm.13454

**Published:** 2024-04-04

**Authors:** Isabelle S. Massart, Axell‐Natalie Kouakou, Nathan Pelet, Pascale Lause, Olivier Schakman, Audrey Loumaye, Michel Abou‐Samra, Louise Deldicque, Laure B. Bindels, Sonia M. Brichard, Jean‐Paul Thissen

**Affiliations:** ^1^ Endocrinology, Diabetes and Nutrition Unit, Institute of Experimental and Clinical Research Université catholique de Louvain Brussels Belgium; ^2^ Institute of Neuroscience Université catholique de Louvain Louvain‐la‐Neuve Belgium; ^3^ Metabolism and Nutrition Research Group, Louvain Drug Research Institute Université catholique de Louvain Brussels Belgium

**Keywords:** adiponectin, cachexia, cancer, inflammation, skeletal muscle

## Abstract

**Background:**

Cancer cachexia is a life‐threatening, inflammation‐driven wasting syndrome that remains untreatable. Adiponectin, the most abundant adipokine, plays an important role in several metabolic processes as well as in inflammation modulation. Our aim was to test whether administration of AdipoRon (AR), a synthetic agonist of the adiponectin receptors, prevents the development of cancer cachexia and its related muscle atrophy.

**Methods:**

The effect of AR on cancer cachexia was investigated in two distinct murine models of colorectal cancer. First, 7‐week‐old CD2F1 male mice were subcutaneously injected with colon‐26 carcinoma cells (C26) or vehicle (CT). Six days after injection, mice were treated for 5 days with AdipoRon (50 mg/kg/day; C26 + AR) or the corresponding vehicle (CT and C26). Additionally, a genetic model, the Apc^*Min*/+^ mouse, that develops spontaneously numerous intestinal polyps, was used. Eight‐week‐old male Apc^*Min/+*^ mice were treated with AdipoRon (50 mg/kg/day; Apc + AR) or the corresponding vehicle (Apc) over a period of 12 weeks, with C57BL/6J wild‐type mice used as controls. In both models, several parameters were assessed in vivo: body weight, grip strength and serum parameters, as well as ex vivo: molecular changes in muscle, fat and liver.

**Results:**

The protective effect of AR on cachexia development was observed in both cachectic C26 and Apc^*Min*/+^ mice. In these mice, AR administration led to a significant alleviation of body weight loss and muscle wasting, together with rescued muscle strength (*P* < 0.05 for all). In both models, AR had a strong anti‐inflammatory effect, reflected by lower systemic interleukin‐6 levels (−55% vs. C26, *P* < 0.001 and −80% vs. Apc mice, *P* < 0.05), reduced muscular inflammation as indicated by lower levels of *Socs3*, phospho‐STAT3 and *Serpina3n*, an acute phase reactant (*P* < 0.05 for all). In addition, AR blunted circulating levels of corticosterone (−46% vs. C26 mice, *P* < 0.001 and −60% vs. Apc mice, *P* < 0.05), the predominant murine glucocorticoid known to induce muscle atrophy. Accordingly, key glucocorticoid‐responsive factors implicated in atrophy programmes were—or tended to be—significantly blunted in skeletal muscle by AR. Finally, AR protected against lipid metabolism alterations observed in Apc^*Min*/+^ mice, as it mitigated the increase in circulating triglyceride levels (−38%, *P* < 0.05) by attenuating hepatic triglyceride synthesis and fatty acid uptake by the liver.

**Conclusions:**

Altogether, these results show that AdipoRon rescued the cachectic phenotype by alleviating body weight loss and muscle atrophy, along with restraining inflammation and hypercorticism in preclinical murine models. Therefore, AdipoRon could represent an innovative therapeutic strategy to counteract cancer cachexia.

## Introduction

Cancer cachexia is a complex metabolic syndrome characterized by weight loss resulting from muscle and fat wasting.^1^ Depending on the type and stage of cancer, 50–80% of all cancer patients are affected by cachexia.^2^ In addition to impairing the quality of life and reducing tolerance to anticancer treatments, cachexia is responsible for at least 20% of all cancer‐related deaths.^2^ Although cancer cachexia is a multi‐organ syndrome, loss of muscle mass in advanced cancer draws particular attention as it is recognized as an independent predictor of mortality.^3^ Interestingly, reversal of muscle loss leads to prolonged survival in animal models of cancer cachexia,^4^, ^5^ supporting that maintaining muscle mass is per se helpful in improving lifespan.

Skeletal muscle loss during cancer results from imbalanced protein homeostasis,^1^ which is largely mediated by inflammation, the main driver of cancer cachexia.^1^ Inflammatory mediators such as proinflammatory cytokines (e.g., interleukin‐6 IL‐6]) and catabolic factors (e.g., glucocorticoids), either derived from tumour‐immune system crosstalk or produced by the tumour itself, activate proteolytic systems and blunt protein synthesis, causing muscle wasting.^1^ In addition, decreased mitochondrial content and function, as well as alterations in myogenesis, may also contribute to muscle atrophy.^6^ To date, efficient pharmacological treatments are still lacking, certainly due to the complexity of this life‐threatening condition.^7^ Although the role of proinflammatory cytokines in cancer cachexia development has been well established, the contribution of anti‐inflammatory mediators (e.g., IL‐10 and adiponectin) has never been investigated.

Adiponectin (ApN) is the most abundant circulating hormone, mainly secreted by adipose tissue. This adipokine exerts insulin‐sensitizing and anti‐inflammatory actions and, hence, is renowned for ameliorating metabolic disorders. ApN exerts its pleiotropic effects via the modulation of several signalling pathways through its two main receptors, AdipoR1 and AdipoR2.^8^ In skeletal muscle, ApN stimulates mitochondrial biogenesis, oxidative metabolism, myogenesis^9^ and muscle regeneration through the AdipoR1/AMP‐activated protein kinase (AMPK)/peroxisome proliferator‐activated receptor γ coactivator‐1α (PGC‐1α) axis. It has been recently shown that ApN has local anti‐inflammatory protective effects in inflammatory myopathies.^10^ In the murine X‐linked muscular dystrophy mouse model in particular, ApN improves muscle function by reducing muscular inflammation and stimulating mitochondrial biogenesis and myogenesis.^11^, ^12^ Moreover, ApN mitigates muscle cell atrophy exposed to proinflammatory cytokines or glucocorticoids.^13^

Despite the pleiotropic and promising properties of ApN on skeletal muscle that could be beneficial in cancer cachexia, its role has been poorly studied so far. Moreover, several studies have shown a reduction of circulating ApN levels in cancer patients as well as in murine models of cachexia.^14^, ^15^, ^16^ Therefore, we hypothesized that restoring ApN signalling could be a promising strategy to counteract cancer cachexia. As the administration of ApN is somewhat challenging because of its complex quaternary structure, rapid turnover and high physiological levels, we used AdipoRon (AR), a small synthetic agonist of AdipoR, which mimics the pleiotropic actions of ApN, including towards skeletal muscle.^17^

In this present study, our aim was to test whether administration of the AdipoR agonist, AdipoRon, prevents the development of cachexia and its related muscle atrophy in two distinct preclinical models of cancer.

## Material and methods

### C26 mouse model of cancer cachexia

After 1‐week acclimatization, 7‐week‐old CD2F1 male mice (Charles River Laboratories, MA, USA) were injected subcutaneously in the upper flank with colon carcinoma C26 cells (1 × 10^6^ cells/0.1 mL of saline; C26 mice) or with saline solution (CT mice). Six days after the injection, three groups of mice with equivalent body weight were formed: CT mice, untreated C26 mice (C26) and C26 mice treated with AdipoRon (C26 + AR). From this day on, mice were injected intraperitoneally daily for 5 days with 50 mg/kg/day of AdipoRon (AdipoGen, San Diego, USA) for the C26 + AR mice group or with the corresponding vehicle (sunflower oil/2.5% dimethyl sulfoxide [DMSO]) for the two other groups. Body weight and food intake were measured daily until euthanasia on Day 11 after the injection of cancer cells.

### Apc^Min/+^ mouse model of cancer cachexia

C57BL/6J Apc^*Min*/+^ male mice, originally purchased from the Jackson Laboratory (JAX #002020) and bred on a C57BL/6J background, were generously given by Damien Freyssenet^18^ and used as a model of colorectal cancer (CRC) cachexia. Seven‐week‐old C57BL/6J Apc^*Min*/+^ and their wild‐type (WT) littermates were habituated to jellified water (Solid Drink Standard, Oud‐Turnhout, Belgium) instead of water bottles for 1 week. At 8 weeks of age, three groups of mice with equivalent body weight were formed: WT mice, untreated Apc^*Min*/+^ mice (Apc) and Apc^*Min*/+^ mice treated with AdipoRon (Apc + AR). For this last group, 50 mg/kg/day of AdipoRon (Tocris, Bristol, UK) was added to jellified water daily for 12 weeks, whereas the two other groups received corresponding vehicles. Body weight and food intake were recorded weekly until euthanasia.

### Ex vivo analyses

Following anaesthesia, serum and tissues (muscles, white adipose tissues [WATs], liver, spleen and tumour polyps) were harvested, frozen in liquid nitrogen and stored at −80°C. The left gastrocnemius (GA) muscle and epididymal white adipose tissue (eWAT) were fixed in 4% formaldehyde. Histological, biochemical and molecular analyses were performed on tissues ex vivo as described in the Supporting Information Methods.

### Statistical analysis

Results are presented as means (± SEM), and the numbers of individuals tested (*n*) are reported in the corresponding figure legends. To assess significant differences between two groups, a two‐tailed unpaired Student's *t*‐test (if parametric) or the Mann–Whitney–Wilcoxon test (if nonparametric) were used. When comparing more than two groups, one‐way analysis of variance (ANOVA) followed by Tukey's post hoc tests or multiple Student's *t*‐tests with Bonferroni's correction (if parametric) or the Kruskal–Wallis global test followed by the Steel–Dwass post hoc test (if nonparametric) were used. If necessary, variables were transformed using binary logarithms before statistical tests to meet the hypothesis of normality (Shapiro–Wilk test). All the statistical analyses were performed using JMP Pro 16 software (SAS Institute Inc., NC, USA) and GraphPad Prism 9 software (San Diego, CA, USA).

## Results

### Cancer is associated with profound drop in circulating adiponectin levels in mice and patients

**Figure 1:**
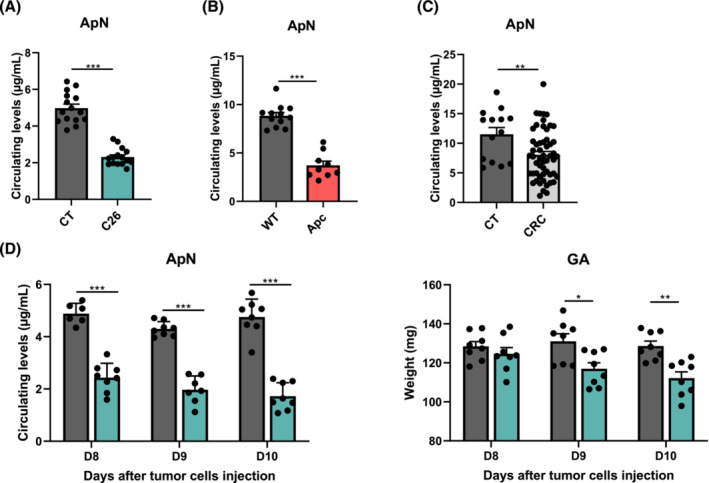
Circulating levels of adiponectin are decreased in mice and in patients with colorectal cancer. (A) Serum adiponectin (ApN) levels in 8‐week‐old CD2F1 male mice injected with C26 colon‐26 carcinoma cells (C26) or corresponding vehicle (CT), 11 days after C26 cell injection (*n* = 15–16 per group). (B) Serum ApN levels in 20‐week‐old male Apc^*Min*/+^ mice (Apc) or wild‐type (WT) mice (*n* = 9–12 per group). (C) Plasma ApN levels in healthy subjects (CT) and colorectal cancer (CRC) patients (CT, *n* = 14; CRC, *n* = 54). (D) Evolution of serum ApN levels and gastrocnemius (GA) muscle weight (as previously reported in Thibaut et al.^19^) in C26 mice or sham‐injected mice (CT) euthanized at 8, 9 and 10 days after C26 cell injection (*n* = 8 per group). Data are reported as mean ± SEM. Significant differences are indicated as *P < 0.05, **P < 0.01 and ***P < 0.001.

We first evaluated the circulating ApN levels associated with CRC. In mice, ApN levels were drastically reduced in the two murine cancer models. Indeed, in the acute C26 model, circulating ApN levels showed a striking drop (−54%) compared with control (CT) mice (*Figure* *1* *A*). Similarly, genetically mutant Apc^*Min*/+^ mice presented a marked reduction in circulating ApN levels (−58%) in comparison with WT mice (*Figure* *1* *B*). Other alterations in the ApN signalling cascade were observed, notably an increased AdipoR1 expression and a decreased AdipoR2 expression in the liver of cancer mice (*Figure* *S1*). In humans, circulating ApN levels were also markedly reduced in CRC patients compared with healthy subjects (CT) (−37%; *Figure* *1* *C*), with no difference between cachectic and non‐cachectic cancer patients (*Figure* *S2*). An equivalent ApN drop was observed in lung cancer patients (*Figure* *S2*).

To unravel the possible contribution of low ApN levels to cancer cachexia, we next investigated the kinetics of the decline in circulating ApN levels during cachexia development in the C26 mouse model. Mice showed a major reduction in ApN levels at Day 8 after inoculation of cancer cells, while weight loss and muscle atrophy (*Figure* *1* *D*) were not yet detectable at this time (pre‐cachexia).^19^

Altogether, these results show that circulating ApN levels are profoundly decreased during cancer and even before significant body weight loss and muscle atrophy.

### AdipoRon alleviates cancer cachexia in C26 mice

**Figure 2:**
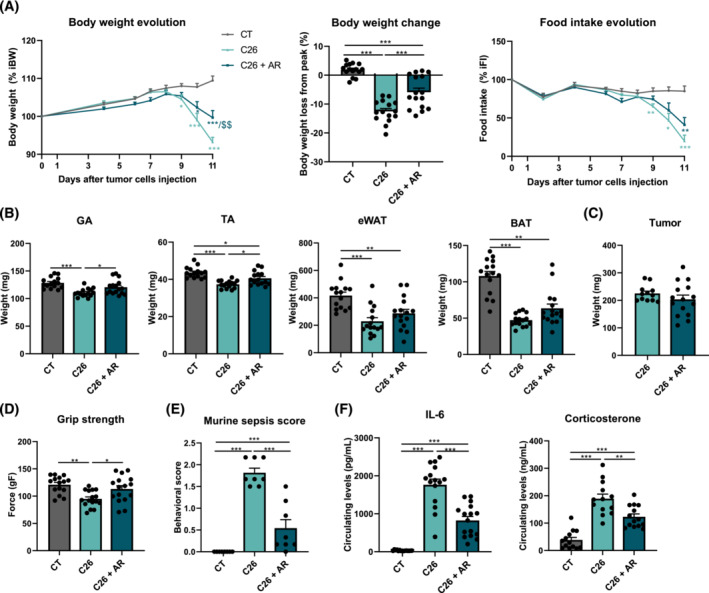
AdipoRon treatment counteracts cancer cachexia by alleviating body weight loss, muscle wasting and inflammation in C26 cachectic mice. Eight‐week‐old CD2F1 male mice were injected with C26 cells (C26) or a vehicle (CT). Six days after cancer cell injection, C26 mice were treated with AdipoRon (AR) intraperitoneally for 5 days (50 mg/kg/day, i.p.; C26 + AR) or corresponding vehicle for the two other groups (CT and C26). (A) Relative changes in body weight and food intake since cancer cell injection (expressed as percentage of initial value) and body weight change between Day 8 (body weight peak in C26 mice) and Day 11, in CT mice (CT), C26 mice (C26) and AR‐treated C26 mice (C26 + AR). (B) Tibialis anterior (TA) muscle, gastrocnemius (GA) muscle, epididymal white adipose tissue (eWAT) and brown adipose tissue (BAT) weights 11 days after cancer cell injection. (C) Tumour weight of C26 and AR‐treated C26 mice at Day 11 (*n* = 13–16 per group). (D) Grip strength (expressed in grams of force) at Day 11. (E) Sepsis score 10 days after cancer cell injection (*n* = 8 per group). (F) Serum IL‐6 and corticosterone levels at Day 11 (*n* = 13–16 per group). Data are reported as mean ± SEM (*n* = 15–16 per group, unless otherwise indicated). Significant differences are indicated as *P < 0.05, **P < 0.01 and ***P < 0.001, or ^$$^ *P* < 0.01 versus C26.

AdipoRon, a synthetic agonist of AdipoR, has shown numerous beneficial effects on muscle metabolism.^17^ To investigate the potential effect of this compound on cancer cachexia, we treated C26 mice with AdipoRon (50 mg/kg/day). Treatment started on Day 5 after C26 cancer cell injection, when the tumour was well established, in order to study the effects of this compound on cachexia progression independently from its potential effect on tumour growth. C26 mice presented a severe loss of body weight mainly due to muscle and adipose tissue wasting, concomitantly with marked anorexia (*Figure* *2* *A*). AdipoRon treatment significantly attenuated the decline in body mass in C26 mice, whereas it had a slight but not significant effect on the drop in food intake (*Figure* *2* *A*). AdipoRon blunted muscle atrophy observed in C26 mice, as reflected by the protected weight of GA and tibialis anterior (TA) muscles in AR‐treated C26 mice (*Figure* *2* *B*). This protective effect on muscle mass was associated with a better muscle function evaluated by the grip strength test, as the marked reduction of strength in C26 mice (−22% vs. CT) was almost completely blunted by AdipoRon (*Figure* *2* *D*). In contrast, the marked fat mass depletion observed in C26 mice (eWAT weight −45% vs. CT) was not significantly rescued by AdipoRon (*Figure* *2* *B*).

Interestingly, AdipoRon exerted its beneficial effects on cancer cachexia without impairing tumour growth, as indicated by the absence of a difference in tumour weight between the two C26 groups (*Figure* *2* *C*). In addition, there was no change in tumour gene expression of proinflammatory cytokines (*Tnfa*, *Il1b*, *Il6* and *Osm*) between untreated and AR‐treated C26 mice (*Figure* *S3*). Importantly, this overall protection of body weight loss with AdipoRon was associated with improvement of general condition, reflected by a lower sepsis score in AR‐treated C26 mice compared with untreated C26 mice (*Figure* *2* *E*). As cancer cachexia is recognized as an inflammation‐driven wasting syndrome and ApN exerts anti‐inflammatory effects, we investigated whether AdipoRon protected against inflammation in C26 mice. As we observed, circulating levels of IL‐6, a major proinflammatory cytokine involved in several models of cachexia, were dramatically increased (∼60‐fold over control levels) in C26 mice and reduced by almost 55% by AdipoRon (*Figure* *2* *F*). Although IL‐6 was clearly expressed by the tumour, its mRNA level was not blunted by AdipoRon (*Figure* *S3*). As the hypothalamo–pituitary–adrenal (HPA) axis is known to be stimulated by inflammatory cytokines and glucocorticoids are recognized as major players in muscle wasting, we evaluated the circulating levels of corticosterone, the predominant murine circulating glucocorticoid. Corticosterone levels were six‐fold higher in C26 mice compared with CT mice and were markedly decreased (−46%) with AdipoRon (*Figure* *2* *F*). Finally, there was no change in ApN circulating levels after AdipoRon treatment (*Figure* *S3*).

### AdipoRon blunts catabolic pathways in skeletal muscle of C26 mice

**Figure 3:**
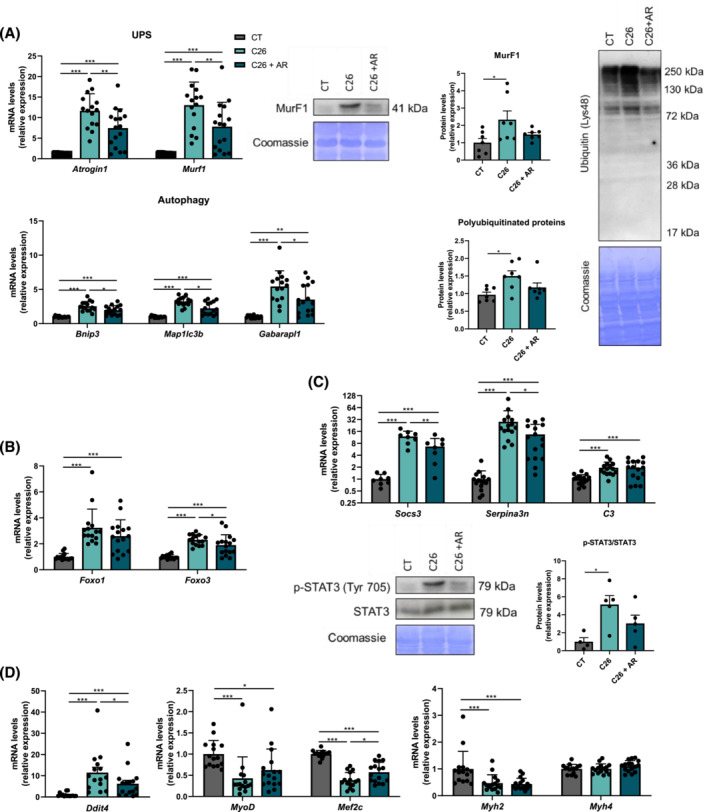
AdipoRon treatment mitigates the muscle activation of the ubiquitin–proteasome system and autophagy in C26 cachectic mice. (A) Relative expression levels of the main genes involved in the ubiquitin–proteasome system (*Atrogin1* and *Murf1*) and autophagy (*Bnip3*, *Map 1lc3b* and *Gabarapl1*) in the tibialis muscle (TA) of sham‐injected mice (CT), untreated C26 mice (C26) and AdipoRon‐injected C26 mice (C26 + AR) 11 days after cancer cell injection. MuRF1 and total poly‐ubiquitinated protein levels measured by western blotting in the skeletal muscle of mice from the three groups. Quantification of protein levels normalized to CT mice (*n* = 7 per group) and representative images. Coomassie blue staining was used as a loading control. (B) Relative gene expression levels of the glucocorticoid‐responsive transcription factors *Foxo1* and *Foxo3* in TA muscle. (C) Relative gene expression levels of *Socs3* and acute phase reactants (*Serpina3n and C3*) in TA muscle. pSTAT3 and STAT3 protein levels measured by western blotting in the skeletal muscle of mice from the three groups. Quantification of pSTAT3/STAT3 protein ratio level normalized to CT mice (*n* = 4–5 per group) and representative images. Coomassie blue staining was used as a loading control. (D) Relative expression levels of genes involved in protein synthesis and/or myogenesis (*Ddit4*, *MyoD*, *Mef2c*, *Myh2* and *Myh4*) in TA muscle. *Atrogin1*, F‐box only protein 32; *Bnip3*, BCL2/adenovirus E1B 19‐kDa protein‐interacting protein 3; *C3*, complement C3; *Ddit4*, DNA damage‐inducible transcript 4 protein; *Foxo1*, forkhead box protein O1; *Foxo3*, forkhead box protein O3; *Gabarapl1*, gamma‐aminobutyric acid receptor‐associated protein‐like 1; *Map 1lc3b*, microtubule‐associated proteins 1A/1B light chain 3B; *Mef2c*, myocyte‐specific enhancer factor 2C; *Murf1*, E3 ubiquitin‐protein ligase TRIM63; *Myh2*, myosin‐2; *Myh4*, myosin‐4; *MyoD*, myoblast determination protein; *Serpina3n*, serine protease inhibitor A3N; *Socs3*, suppressor of cytokine signalling 3. Data are reported as mean ± SEM (*n* = 15–16 per group, unless otherwise indicated). Significant differences are indicated as *P < 0.05, **P < 0.01 and ***P < 0.001.

In order to better understand the mechanisms of muscle mass protection provided by AdipoRon, we investigated its impact on catabolic pathways in skeletal muscle. Because AdipoRon reduced circulating IL‐6 levels, together with corticosterone, we speculated that a decrease in catabolic processes induced by inflammation and glucocorticoids, namely, the two main proteolytic systems, the ubiquitin–proteasome and autophagy–lysosomal systems, could contribute to its anti‐atrophic effect. In accordance with this hypothesis, we showed a marked alleviation of the induction of atrogenes, *Murf1* and *Atrogin1*, and autophagy‐related genes *Bnip3*, *Map 1lc3b* and *Gabarapl1* in muscle from AR‐treated C26 mice compared with C26 mice (*Figure* *3* *A*). In line with mRNA atrogene regulation, upregulated levels of poly‐ubiquitinated proteins and MuRF1 protein observed in C26 mice were blunted by AdipoRon (*Figure* *3* *A*). Consistent with these findings, we observed with AdipoRon a reduced expression of glucocorticoid‐responsive transcription factors, *Foxo1/3* (*Figure* *3* *B*), of an IL‐6 dependent gene, *Socs3*, related to decreased pSTAT3 (*Figure* *3* *C*), of components of the non‐canonical nuclear factor kappa B (NF‐κB) pathway (*Nik*, *Nfkb2* and *Relb*) (*Figure* *S4*) and of some acute phase reactants (APRs) (*Serpina3n*), which testifies that the muscle was less exposed to glucocorticoids and inflammatory stimuli, respectively.

As reduced anabolic signalling also contributes to muscle atrophy in cancer cachexia, we tested the impact of AdipoRon on REDD‐1 expression (*Ddit4*), an inhibitor of the mammalian target of rapamycin complex 1 (mTORC1), which is under the positive transcriptional control of glucocorticoids. The dramatic induction of *Ddit4* expression in the muscles of C26 mice was significantly blunted by AdipoRon (*Figure* *3* *D*). However, the decrease in ribosomal mass as assessed by rRNA content was not affected by AR (*Figure* *S5*), which does not argue for a stimulatory effect of AR on protein synthesis. We reported in parallel a decrease in myogenic transcription factor's expression (*MyoD* and *Mef2c*), which was—or tended to be—prevented by AdipoRon (*Figure* *3* *D*). Moreover, expression of myosin heavy chains was either unchanged (*Myh4*) or similarly reduced (*Myh2*) in AR‐treated and AR‐untreated C26 mice (*Figure* *3* *D*). Finally, AdipoRon prevented or tended to prevent the decrease of mitochondrial transcriptional regulator gene expression, *Esrra*, and mitochondrial DNA content, suggesting stimulation of mitochondrial biogenesis through the AMPK/PGC‐1α axis by AdipoRon (*Figure* *S6*).

### AdipoRon alleviates cancer cachexia in Apc^*Min*/+^ mice

In order to strengthen the results observed in C26 mice, we administered AdipoRon to Apc^*Min*/+^ mice (50 mg/kg/day) for 12 weeks. Compared with C26 mice, these mice, which develop spontaneously multiple intestinal neoplasia, present more progressive skeletal muscle and adipose tissue wasting in response to the tumour, hence mimicking more closely the clinical condition of human cancer cachexia.

**Figure 4:**
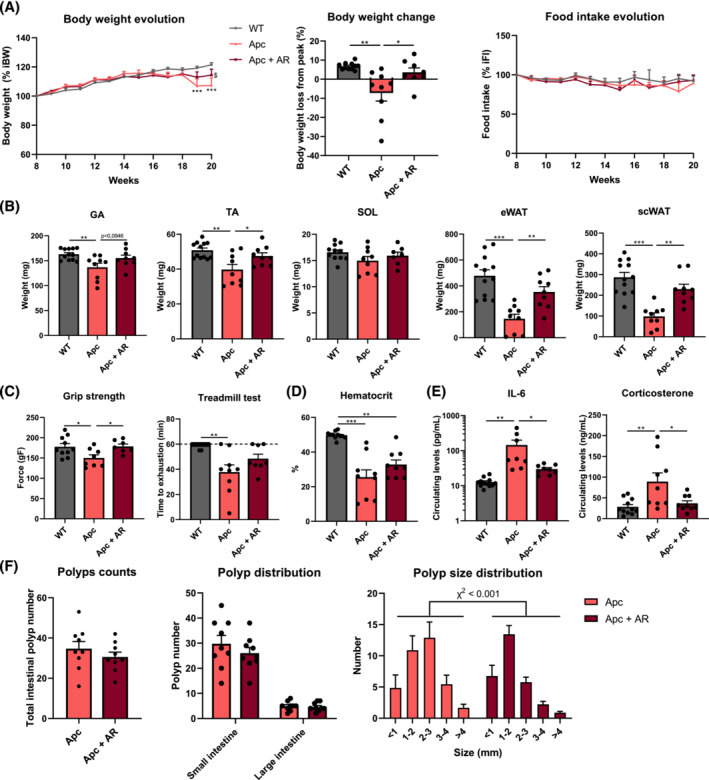
AdipoRon treatment counteracts cancer cachexia by alleviating body weight loss, muscle and adipose tissue wasting and inflammation in Apc^*Min*/+^ cachectic mice. (A) Relative changes in body weight and food intake since Week 8 (expressed as percentage of initial value) and body weight change between Week 15 (body weight peak in Apc^*Min*/+^ mice) and Week 20 in C57BL/6J wild‐type (WT) mice, Apc^*Min*/+^ mice (Apc) and Apc^*Min*/+^ mice receiving AdipoRon (AR) in their water (Apc + AR). (B) Tibialis anterior (TA), gastrocnemius (GA), soleus (SOL) muscles, epididymal (eWAT) and subcutaneous (scWAT) white adipose tissue weights at 20 weeks. (C) Grip strength (expressed in grams of force) at 18 weeks and treadmill test at 15 weeks (*n* = 7–12 per group). The maximum test duration was set at 60 min. (D) Haematocrit levels at 20 weeks. (E) Circulating IL‐6 and corticosterone levels at 20 weeks (*n* = 8–12 per group). (F) Total intestinal polyp number in the small intestine and colon, polyp intestinal distribution and polyp size distribution of Apc and Apc + AR mice at 20 weeks (*n* = 9 per group). Data are reported as mean ± SEM (*n* = 9–12 per group, unless otherwise indicated). Significant differences are indicated as *P < 0.05, **P < 0.01 and ***P < 0.001, or ^$^ *P* < 0.05 versus Apc.

Compared with WT mice, Apc^*Min*/+^ mice exhibited a significant loss of body weight from Week 18 (*Figure* *4* *A*), and the daily administration of AdipoRon prevented this wasting, which became significantly different at 20 weeks of age (*Figure* *4* *A*). In contrast to C26 mice, Apc^*Min*/+^ mice did not present anorexia (*Figure* *4* *A*), hence making *per os* treatment achievable. Importantly, AdipoRon treatment did not affect daily or cumulative food intake (*Figure* *S7*).

As expected, Apc^*Min*/+^ mice exhibited marked muscle atrophy after 20 weeks, as indicated by reduced GA and TA weights compared with WT mice (*Figure* *4* *B*). Along with the protection of body weight loss, AdipoRon prevented or tended to prevent muscle mass loss, as suggested by the attenuated mass loss of GA and TA muscles. No difference in the soleus (SOL) muscle weight was observed between the three groups. Furthermore, adipose tissue mass, reflected by the weight of subcutaneous white adipose tissue (scWAT)/eWAT, was strikingly reduced in Apc^*Min*/+^ mice compared with WT mice, while AdipoRon rescued this depletion (*Figure* *4* *B*).

To determine the functional consequences of AdipoRon treatment, we next evaluated the effect of AdipoRon on physical performance. First, we evaluated muscle force by using a grip strength test. Apc^*Min*/+^ mice presented a significant reduction of their tension force (−16% vs. WT), which was, in line with its protective effects on muscle mass, thwarted by AdipoRon (*Figure* *4* *C*). In addition, we assessed the effect of AdipoRon on muscle endurance by using a treadmill test. Endurance capacity was markedly decreased in both Apc^*Min*/+^ groups, with a slight but not significant protective effect of AdipoRon (*Figure* *4* *C*). As muscle endurance depends on oxidative metabolism, we assessed mitochondrial mass and the haematocrit as markers of oxygen availability. Both parameters were decreased in Apc^*Min*/+^ and were not—or only partially—reversed by AdipoRon (*Figures* *S6* and *4* *D*).

Consistent with our findings in C26 mice, circulating IL‐6 levels were also markedly induced (~11‐fold) in Apc^*Min*/+^ mice, and AdipoRon blunted this induction by 80% (*Figure* *4* *E*). To identify the sources of circulating IL‐6, we measured *Il6* mRNA levels in different tissues. IL‐6 was markedly induced in several tissues, and AdipoRon blunted these inductions, suggesting a systemic anti‐inflammatory effect of AdipoRon (*Figure* *S7*). Moreover, circulating corticosterone levels were increased by ~3‐fold in Apc^*Min*/+^ mice, while AdipoRon mitigated this increase by almost 60% (*Figure* *4* *E*). AdipoRon protected slightly against the decrease in ApN circulating levels (*Figure* *S7*).

Finally, we examined if polyp burden in Apc^*Min*/+^ mice was affected by AdipoRon. Total polyp number, predominantly located in the small intestine, was unaffected by AdipoRon (*Figure* *4* *F*). However, tumoral gene expression of cytokines tended to decrease (*Figure* *S7*), while polyp size distribution was significantly different (*P* < 0.001) between Apc and Apc + AR mice, with the number of polyps of 2–3 mm in diameter being reduced by ~55% in Apc + AR mice.

Collectively, our data indicate that AdipoRon exerts a strong protective effect on cachexia development with attenuation of muscle wasting in cachectic C26 and Apc^*Min*/+^ mice.

### AdipoRon blunts catabolic pathways in skeletal muscle of Apc^*Min*/+^ mice

**Figure 5:**
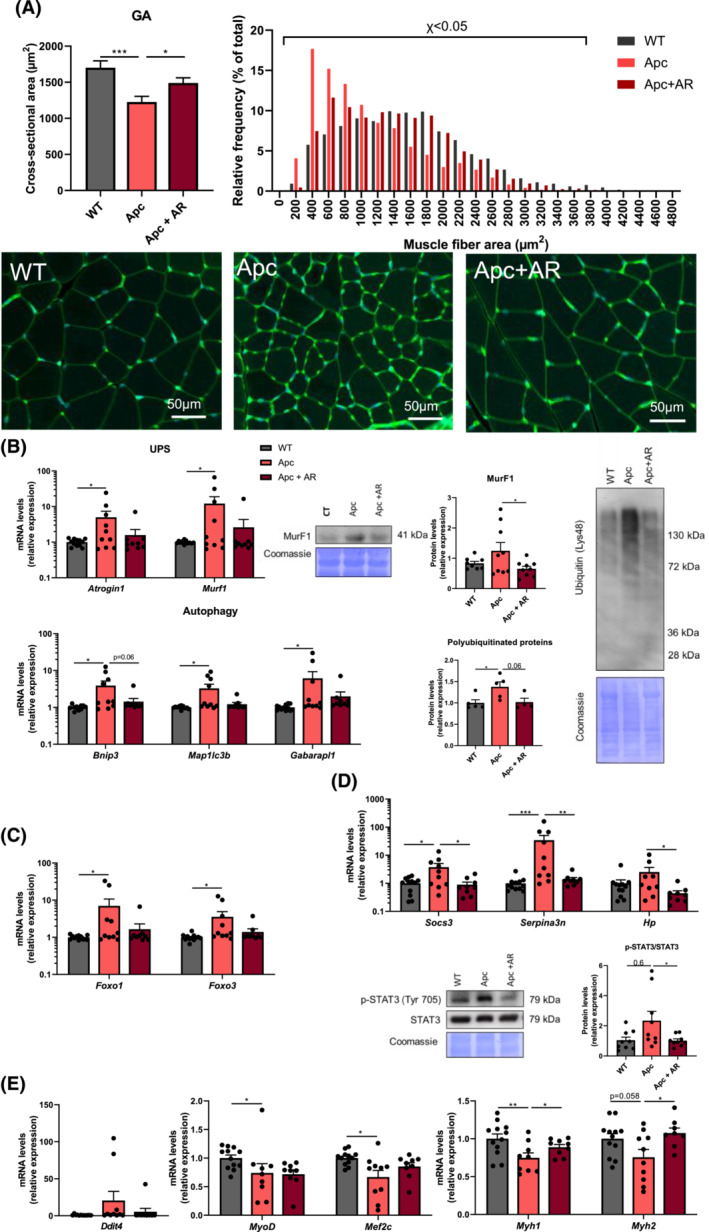
AdipoRon treatment mitigates the muscle activation of the ubiquitin–proteasome system and autophagy in Apc^*Min*/+^ cachectic mice. (A) Mean cross‐sectional area of gastrocnemius (GA) muscle fibre and relative frequency distribution (percentage to total fibre number) of GA muscle fibre cross‐sectional area of C57BL/6J wild‐type (WT) mice, Apc^*Min*/+^ mice (Apc) and Apc^*Min*/+^ mice receiving AdipoRon in their water (Apc + AR) at 20 weeks. Representative images of GA muscle sections stained with rhodamine‐labelled WGA (wheat germ agglutinin) for each group are shown. Scale bar =  50 μm. (B) Relative expression levels of main genes involved in the ubiquitin–proteasome system (*Atrogin1* and *Murf1*) and autophagy (*Bnip3*, *Map 1lc3b* and *Gabarapl1*) in GA muscle. MuRF1 and total poly‐ubiquitinated protein levels measured by western blotting in the skeletal muscle of mice from the three groups. Quantification of protein level normalized to WT mice (*n* = 4–9 per group) and representative images. Coomassie blue staining was used as a loading control. (C) Relative gene expression levels of the glucocorticoid‐responsive transcription factors *Foxo1* and *Foxo3* in GA muscle. (D) Relative gene expression levels of *Socs3* and inflammatory acute phase reactant proteins (*Serpina3n* and *Haptoglobin*) in GA muscle. pSTAT3 and STAT3 protein levels measured by western blotting in the skeletal muscle of mice from the three groups. Quantification of pSTAT3/STAT3 protein ratio level normalized to CT mice (*n* = 9 per group) and representative images. Coomassie blue staining was used as a loading control. (E) Relative expression levels of genes involved in protein synthesis and/or myogenesis (*Ddit4*, *MyoD*, *Mef2c* and *Myh1/2*) in GA muscle. *Atrogin1*, F‐box only protein 32; Bnip3, BCL2/adenovirus E1B 19‐kDa protein‐interacting protein 3; *Ddit4*, DNA damage‐inducible transcript 4 protein; *Foxo1*, forkhead box protein O1; *Foxo3*, forkhead box protein O3; *Gabarapl1*, gamma‐aminobutyric acid receptor‐associated protein‐like 1; Hp, haptoglobin; *Map 1lc3b*, microtubule‐associated proteins 1A/1B light chain 3B; *Mef2c*, myocyte‐specific enhancer factor 2C; *Murf1*, E3 ubiquitin‐protein ligase TRIM63; *Myh1*, myosin‐1; *Myh2*, myosin‐2; *MyoD*, myoblast determination protein; *Serpina3n*, serine protease inhibitor A3N; *Socs3*, suppressor of cytokine signalling 3. Data are reported as mean ± SEM (*n* = 9–12 per group, unless otherwise indicated). Significant differences are indicated as *P < 0.05, **P < 0.01 and ***P < 0.001.

The decrease in GA weight in Apc^*Min*/+^ mice (*Figure* *4* *B*) was associated with decreased muscle fibre size, while AdipoRon alleviated this decrease (*Figure* *5* *A*). In order to investigate the mechanisms of this protective effect on muscle fibre atrophy, we quantified the expression of genes involved in the two major proteolytic systems. We showed that these two systems are activated in the muscle of Apc^*Min*/+^ mice, as reflected by the marked induction of *Murf1*, *Atrogin1*, *Bnip3*, *Map 1lc3b* and *Gabarapl1*, whereas AdipoRon almost abolished their induction (*Figure* *5* *B*). As in C26 mice, the upregulated levels of poly‐ubiquitinated proteins and MuRF1 protein observed in Apc mice were attenuated by AdipoRon (*Figure* *5* *B*). In accordance with these findings, we observed with AdipoRon a reduced expression of glucocorticoid‐responsive transcription factors, *Foxo1/3* (*Figure* *5* *C*), of the IL‐6 dependent gene, *Socs3*, related to decreased pSTAT3 (*Figure* *5* *D*), of components of the non‐canonical NF‐κB pathway (*Nik*, *Nfkb2* and *Relb*) (*Figure* *S4*) and of some APRs (*Serpina3n*) (*Figure* *5* *D*) in Apc^*Min*/+^ mice, consistent with its anti‐inflammatory effect.

We also investigated the expression of genes involved in muscle anabolism. First, as observed in C26 mice, the expression of REDD‐1 (*Ddit4*) tended to be induced in the muscle of cachectic mice and reduced by AdipoRon (*Figure* *5* *E*). However, ribosomal mass as assessed by rRNA content was not different between the three groups (*Figure* *S5*). We did not observe any protective effects of AdipoRon on the drop in expression of pro‐myogenic factors (*Myod* and *Mef2c*) in Apc^*Min*/+^ mice (*Figure* *5* *E*). By contrast, the drop in expression of fast‐type myosin heavy chains *Myh1* and *Myh2* in Apc^*Min*/+^ mice was significantly rescued by AdipoRon (*Figure* *5* *E*). Finally, AdipoRon prevented or tended to prevent the decrease of mitochondrial transcriptional regulator gene expression, *Esrra*, and mitochondrial DNA content, suggesting a stimulation of mitochondrial biogenesis through the AMPK/PGC‐1α axis (*Figure* *S6*).

Taken together, these findings indicate that AdipoRon protected against muscle wasting in both cachectic C26 and Apc^*Min*/+^ mice, and this effect might be predominantly mediated through an anti‐proteolytic effect.

### Impaired lipid metabolism in cachectic Apc^*Min*/+^ mice is alleviated by AdipoRon

**Figure 6:**
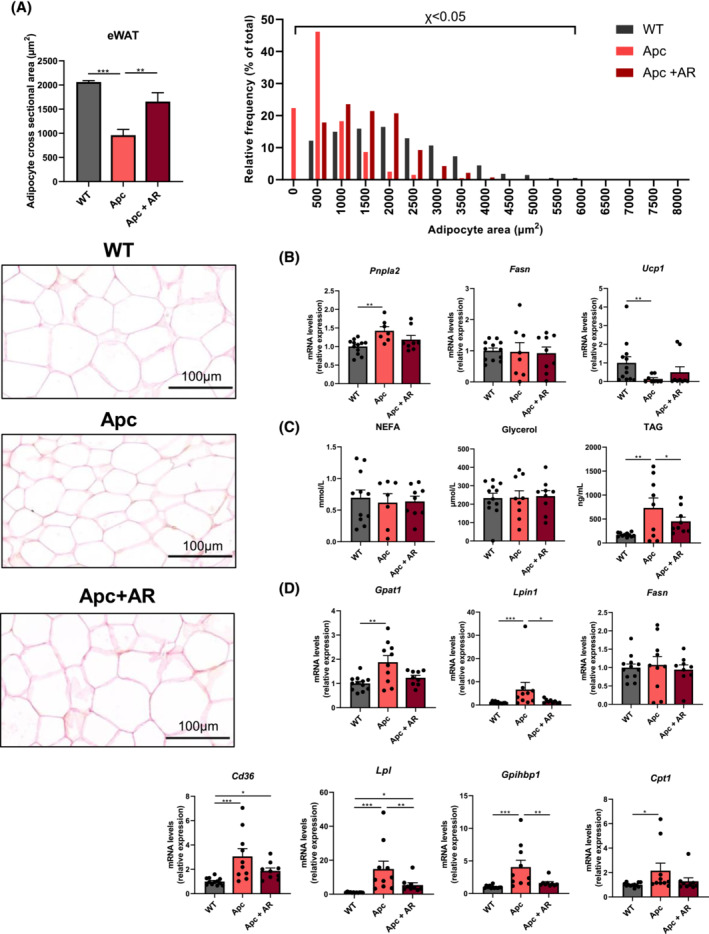
AdipoRon reverses lipid metabolism alterations in Apc^*Min*/+^ cachectic mice. (A) Mean cross‐sectional area of adipocytes in epididymal white adipose tissue (eWAT) and relative frequency distribution (percentage of total adipocyte number) of adipocytes cross‐sectional area in C57BL/6J wild‐type (WT) mice, Apc^*Min*/+^ mice (Apc) and Apc^*Min*/+^ mice receiving AdipoRon in their water (Apc + AR) at 20 weeks. Representative images of white adipose tissue sections stained with haematoxylin–eosin (HE) for each group are shown. Scale bar = 100 μm. (B) Subcutaneous white adipose tissue gene expression levels of enzymes involved in lipolysis (*Pnpla2*), de novo lipogenesis (*Fasn*) and browning (*Ucp1*). (C) Serum levels of total non‐esterified fatty acids (NEFAs), glycerol and triglycerides (TAG) (*n* = 7–12 per group). (D) Hepatic gene expression levels of enzymes involved in triglyceride synthesis (*Gpat1* and *Lpin1*), de novo lipogenesis (*Fasn*), fatty acid uptake (*Cd36*), triglyceride hydrolysis (*Lpl* and *Gpihbp1*) and fatty acid β‐oxidation (*Cpt1a*). *Cd36*, platelet glycoprotein 4; *Cpt1a*, carnitine *O*‐palmitoyltransferase 1, liver isoform; *Fasn*, fatty acid synthase; *Gpat1*, glycerol‐3‐phosphate acyltransferase 1, mitochondrial; *Gpihbp*1, glycosylphosphatidylinositol‐anchored high‐density lipoprotein‐binding protein 1; *Lpin1*, phosphatidate phosphatase LPIN1; *Lpl*, lipoprotein lipase; *Pnpla2*, patatin‐like phospholipase domain‐containing protein 2; *Ucp1*, mitochondrial brown fat uncoupling protein 1. Data are reported as mean ± SEM (*n* = 9–12 per group, unless otherwise indicated). Significant differences are indicated as *P < 0.05, **P < 0.01 and ***P < 0.001.

As shown earlier, AdipoRon strikingly alleviated fat mass loss in Apc^*Min*/+^ mice (*Figure* *4* *B*). This protection was associated with the rescue of reduced adipocyte area by AdipoRon (*Figure* *6* *A*). In order to investigate this striking protective effect, we evaluated the transcript levels of the main enzymes involved in lipolysis and de novo lipogenesis in WAT. The expression of the rate‐limiting lipase, *Pnpla2*, a.k.a. as *Atgl*, was induced in WAT from Apc^*Min*/+^ mice (+42% vs. WT), while AdipoRon tended to decrease this induction (*Figure* *6* *B*). Regarding lipogenesis, *Fasn* was similarly expressed within the three groups. The marked decrease of *Upc1* mRNA levels in both Apc groups suggests that increased WAT browning was not involved in fat mass depletion (*Figure* *6* *B*).

To gain deeper insight into the mechanisms involved in fat loss, we analysed the circulating levels of products derived from lipid metabolism. While non‐esterified fatty acids (NEFAs) and glycerol serum levels were unchanged within the three groups (*Figure* *6* *C*), severe hypertriglyceridaemia was observed in Apc^*Min*/+^ mice (by ~4.3‐fold vs. WT; *Figure* *6* *C*) and was attenuated significantly by AdipoRon (−38% vs. Apc). As triglycerides are mainly produced by the liver, we hypothesized that hepatic lipid metabolism could be altered in Apc^*Min*/+^ mice. Therefore, we investigated the gene expression levels of key hepatic enzymes involved in lipid metabolism. First, we explored hepatic triglyceride production by measuring the expression of key enzymes involved in triglyceride synthesis, *Gpat1* and *Lpin1*, which were increased in Apc^*Min*/+^ mice and normalized by AdipoRon (*Figure* *6* *D*). This enhanced triglyceride synthesis could result from higher hepatic fatty acid synthesis or uptake into the liver. As in adipose tissue, *Fasn* mRNA levels were similar between the three experimental groups, indicating that de novo lipogenesis was not increased in the liver of Apc^*Min*/+^ mice. In contrast, we reported a marked increase in *Cd36* gene expression, a mediator of fatty acid uptake, that AdipoRon tended to counteract (*Figure* *6* *D*). In addition to fatty acids released by adipose tissue, fatty acids taken by the liver could be raised by the enhanced extracellular lipolysis of circulating triglycerides, as suggested by the increased expression of *Lpl* and *Gpihbp1* that is involved in Lpl translocation to the capillary lumen that AdipoRon both significantly reduced. Finally, fatty acids released from adipose tissue could be catabolized by increased hepatic β‐oxidation, as evidenced by the induction of *Cpt1*, which tended to be rescued by AdipoRon (*Figure* *6* *D*).

Altogether, these data indicate that AdipoRon attenuates fat depletion and improves key markers of lipid metabolism in Apc^*Min*/+^ mice.

## Discussion

To test the hypothesis that ApN signalling activation could alleviate cancer cachexia, we performed an interventional study by administering AdipoRon, a synthetic agonist of ApN receptors, in two distinct murine models of cancer cachexia, one acute, the C26 mice, and one subacute, the Apc^*Min*/+^ mice. The major findings are that, in response to AdipoRon, cachectic mice in the two models are protected against the main pathological features of cancer cachexia, in particular body weight loss, muscle wasting and muscle strength loss. Our results suggest, therefore, that the decline in circulating ApN levels that we observed in cancer may contribute to cancer cachexia.

According to our results, the anti‐atrophic effect of AdipoRon results at least in part from inhibition of muscle proteolysis, as indicated by downregulation of the two main proteolytic systems, namely, the ubiquitin–proteasome and autophagy systems.^2^ Possibly, increased mitochondrial biogenesis and myogenesis may also play a role, as suggested by our observations. However, it is worth noting the absence of any stimulatory effect of AdipoRon on molecular pathways driving muscle protein synthesis (data not shown). Besides protecting muscle mass, AdipoRon also preserved muscle strength, which can certainly be related to preserved mass but also to a change in fibre phenotype. Indeed, the decrease in expression of type II‐fast muscle fibres, expressing *Myh1* and *Myh2*, which produce the highest power and are the most affected by cancer cachexia,^20^ is counteracted by AdipoRon in Apc^*Min*/+^ mice. Similar to previous findings,^21^, ^22^ we observed enhanced fatigability in Apc^*Min*/+^ mice on treadmill tests. Although it was reported that AdipoRon improves muscle oxidative capacity by increasing, among others, mitochondrial content,^23^ as we observed, this was not associated with rescued endurance in AR‐treated Apc^*Min*/+^ mice. These unexpected results could be due to persistent anaemia leading to inadequate oxygen delivery to muscle and therefore altering oxidative capacity and fatigue resistance.

The protective effect exerted by AdipoRon on muscle mass and function was most likely mediated through inhibition of cachexia‐associated inflammation, as supported by several observations. First, the induction of IL‐6, the main inflammatory driver of cachexia, was markedly blunted by AdipoRon consistently in the two animal models. The anti‐cachectic effects of AdipoRon through IL‐6 inhibition are in accordance with previous studies. Indeed, administration of anti‐IL‐6 or anti‐IL‐6 receptor antibodies counteracted body weight loss in C26 mice^24^ and in Apc^*Min*/+^ mice,^25^ as well as in other models.^26^ Along with body weight protection, IL‐6 signalling inhibition in cancer cachexia protected against muscle wasting by attenuating proteolytic systems.^25^ Second, the downregulation of muscle p‐STAT3 levels and *Socs3* expression in response to AdipoRon is indicative of a decreased activation of the IL‐6/STAT3 signalling in muscle, an intracellular pathway involved in muscle atrophy. Indeed, targeted‐muscle STAT3 signalling suppression protected against atrophy in tumour‐bearing mice.^27^, ^28^ Third, the inflammation‐induced production of APRs in cachectic mice, as indicated by *Serpina3n* induction in muscle and liver, was blunted by AdipoRon. We previously showed that muscle APR production, which requires mobilization of amino acids coming from muscle proteolysis, was associated with muscle atrophy in cachexia preclinical models, and it was negatively correlated with muscularity in cachectic cancer patients.^29^ Therefore, by decreasing APR synthesis, AdipoRon could indirectly protect against protein catabolism. Finally, AdipoRon hindered muscular gene expression of components of the non‐canonical NF‐κB pathway that is involved in atrogene induction during cachexia.^30^

Inhibition of systemic inflammation by AdipoRon may also indirectly preserve muscle mass in cancer cachexia. Indeed, systemic inflammation observed in cancer is commonly associated with activation of the HPA axis and subsequent release of glucocorticoids.^31^ Previous studies have pinpointed the crucial role of glucocorticoids in C26 and Apc^*Min*/+^ models of cachexia.^18^, ^32^ More specifically, it has been shown that muscle‐specific deletion of the glucocorticoid receptor attenuates cachexia progression and muscle atrophy.^32^ Therefore, by indirectly blunting HPA activation, AdipoRon could also exert its anti‐atrophic effects, a hypothesis that is supported by several observations. First, similarly to previous findings,^18^, ^33^ we observed in our two models of cachexia an increase in circulating corticosterone levels, which was alleviated by AdipoRon. Second, the induction of catabolic glucocorticoid‐responsive genes, in particular transcriptional factors such as Foxo1/3, or enzymes involved in proteolytic systems, was reduced by AdipoRon. Therefore, by blunting circulating corticosterone induction and its downstream signalling in the muscles of cachectic mice, AdipoRon could protect against muscle mass loss.

It is well established that activation of ApN receptors leads to the activation of the AMPK pathway, a key energy sensor and regulator of metabolic homeostasis.^8^ In cancer, pharmacological AMPK activation has been reported to protect against cachexia progression, as administration of the AMPK activator, 5‐aminoimidazole‐4‐carboxamide ribonucleotide (AICAR), prevented body weight loss, together with reduced muscle wasting and induction of the atrogenes *Atrogin1* and *Murf1* in cachectic tumour‐bearing mice.^34^, ^35^ These findings may be surprising, as the majority of studies reported an increase in AMPK activation in muscle from cachectic mice in both C26 and Apc^*Min*/+^ models, which seems rather deleterious, notably by contributing to anabolic suppression via AMPK/mTOR signalling.^25^ This inconsistency could be explained by the fact that activated muscle AMPK is reported in the late state of cachexia, while it has been shown that AMPK activation could be beneficial before the onset of cachexia symptoms. Our data substantiate these findings as we intervened with AdipoRon before the onset of cachexia in these two models. Nevertheless, the role of AMPK in muscle atrophy is still disputed, as muscle‐specific deletion of AMPK has been reported to protect^36^ or exacerbate^37^ cachexia. In contrast to muscle, AMPK expression and activity have been reported to be reduced in WAT from C26 and Apc^*Min*/+^ mice, while AMPK‐stabilizing peptide ameliorated cachectic conditions by attenuating fat depletion.^38^ Therefore, our data strengthened the previous findings that targeting AMPK can be a tempting therapeutic perspective for alleviating cachexia, although further studies are needed to delineate its precise role.

The anti‐cachectic effect of AdipoRon is not limited to skeletal muscle but also extends to adipose tissue. Indeed, the major fat depletion observed in Apc^*Min*/+^ mice was almost fully reversed by AdipoRon. According to our results, the protective effect of AdipoRon towards fat mass may result from attenuation of lipolysis in adipose tissue together with decreased fatty acid uptake associated with reduced triglyceride synthesis by the liver. Indeed, AdipoRon tended to reduce the expression of adipose triglyceride lipase (*Pnpla2*), the rate‐limiting enzyme of lipolysis in adipose tissue, and to alleviate the enhanced hepatic fatty acid uptake and triglyceride synthesis by the liver, as indicated by lower *Cd36* and *Gpat1* expression, respectively, therefore contributing to lowering hypertriglyceridaemia. Consistent with this hypothesis, it has been shown that hepatic deletion of *Gpat1*, the enzyme catalysing the first rate‐limiting step in triglyceride synthesis, leads to lower hepatic triglyceride content, together with reduced very low‐density lipoprotein (VLDL) secretion rate and plasma triglyceride levels.^39^ Conversely, overexpression of *Gpat1* in the liver results in increased triglyceride synthesis and subsequent hepatic steatosis, even in the absence of overnutrition.^40^ Interestingly, liver *Gpat1* has been shown to be downregulated via the AMPK pathway.^41^ Knowing that AdipoRon could activate the AMPK pathway in the liver^8^ supports our interpretation. Taken together, these data suggest that AdipoRon improves whole‐body lipid metabolism by preventing excessive synthesis of hepatic triglycerides from fatty acids derived from adipose tissue.

Altogether, our results demonstrate a protective effect of AdipoRon against cachexia in two preclinical models of CRC, as it rescues the cachectic phenotype by markedly alleviating body weight loss and muscle atrophy, along with restraining inflammation. Therefore, AdipoRon, and more generally, AdipoR agonists, could represent an innovative therapeutic strategy to counteract cancer cachexia, which deserves further exploration.

## Conflict of interest statement

The authors declare no conflict of interest.

## Funding

This work was funded by the Télévie from the Fonds de la Recherche Scientifique ‐ FNRS under Grants 7450318 and 7652020.

## Supporting information


**Table S1.** Criteria for murine sepsis score determination.
**Table S2.** List of primer sequences used for real‐time quantitative PCR analysis.
**Table S3.** Characteristics of healthy subjects and cancer patients.
**Figure S1.** Effects of AdipoRon on Adiponectin receptor (*AdipoR1* and *AdipoR2*) gene expression in cachectic C26 and Apc^*Min*/+^ mice.
**Figure S2.** Adiponectin levels are reduced during cancer.
**Figure S3.** Effects of AdipoRon on adiponectin levels, tumoral gene expression and tissue IL‐6 expression in cachectic C26 mice.
**Figure S4.** Effects of AdipoRon on canonical and non‐canonical NF‐KB pathways in cachectic C26 and Apc^*Min*/+^ mice.
**Figure S5.** Effects of AdipoRon on ribosomal RNA content in skeletal muscle of cachectic C26 and Apc^*Min*/+^ mice.
**Figure S6.** Effects of AdipoRon on gene expression of *Esrra*, a transcriptional regulator of mitochondrial biogenesis, and on mitochondrial content in skeletal muscle of cachectic C26 and Apc^*Min*/+^ mice.
**Figure S7.** Effects of AdipoRon on food intake, adiponectin levels, polyp gene expression and tissue IL‐6 expression in cachectic Apc^*Min*/+^ mice.
