# Supplementary material for: Administration of adiponectin receptor agonist AdipoRon relieves cancer cachexia by mitigating inflammation in tumour‐bearing mice
Source: J Cachexia Sarcopenia Muscle. 2024 Apr 4;15(3):919–33. doi: 10.1002/jcsm.13454 (PMC11154773; doi:10.1002/jcsm.13454)
Supplement: Supplementary file 1 — Table S1. Criteria for murine sepsis score determination. Table S2. List of primer sequences used for real‐time quantitative PCR analysis. Table S3. Characteristics of healthy subjects and cancer patients. Figure S1. Effects of AdipoRon on Adiponectin receptor (AdipoR1 and AdipoR2) gene expression in cachectic C26 and Apc Min/+ mice. Figure S2. Adiponectin levels are reduced during cancer. Figure S3. Effects of AdipoRon on adiponectin levels, tumoral gene expression and tissue IL‐6 expression in cachectic C26 mice. Figure S4. Effects of AdipoRon on canonical and non‐canonical NF‐KB pathways in cachectic C26 and Apc Min/+ mice. Figure S5. Effects of AdipoRon on ribosomal RNA content in skeletal muscle of cachectic C26 and Apc Min/+ mice. Figure S6. Effects of AdipoRon on gene expression of Esrra, a transcriptional regulator of mitochondrial biogenesis, and on mitochondrial content in skeletal muscle of cachectic C26 and Apc Min/+ mice. Figure S7. Effects of AdipoRon on food intake, adiponectin levels, polyp gene expression and tissue IL‐6 expression in cachectic Apc Min/+ mice. [file JCSM-15-919-s001.pdf]

SUPPORTING INFORMATION

Table of content

Supplementary methods 2

Supplementary tables 8

Supplementary figures 12

Supplementary references 20

SUPPLEMENTARY METHODS

Cell culture

Colon carcinoma C26 cells, provided by Dr Mario Colombo [1], were cultured in Dulbecco's modified Eagle medium (DMEM) high glucose supplemented with 10% fetal bovine serum (PAA clone, Pasching, Austria), 100 µg/ml streptomycin and 100 IU/ml penicillin (Gibco, Inchinnan, Scotland) at 37°C with 5% CO_2.

Animals

All the mice were group housed (2-3 per cage) under a 12h light-dark cycle and fed with standard rodent chow diet *ad libitum* (AO4-10, SAFE, Augy, France). All mouse experiment protocols were approved by the ethical committee for animal care of the Health Sector of the Université catholique de Louvain (Brussels, Belgium) under the reference 2018/UCL/MD/033 and 2017/UCL/MD/005. In C26 mice, disease severity was assessed using a Murine Sepsis Score scoring system, adapted from [6] (Table S1 in Supplementary Materials). The sepsis score was calculated as the average of the scores for six components listed in Table S1: appearance, level of consciousness, activity, response to stimulus, eyes and respiratory quality (Supplementary tables).

AdipoRon administration

The daily dose of 50 mg /kg/day of AdipoRon was chosen basis on studies showing that plasma AdipoRon reaches the maximum concentration (11.8 µM) 2 h after oral gavage at 50 mg/kg, a value higher that the affinity of AdipoR1 and R2 for AdipoRon, which is respectively 1.9 µM and 3.1 µM [2]. Similarly, Onodera et al. (2021) showed that after IP injection of AdipoRon at 20 mg/kg, circulating concentration peaks at 5 µM.

Oral route was chosen to administer AdipoRon in Apc mice. However, this route was not suitable in C26 mice since they develop marked anorexia that could prevent adequate intake of the drug. For this reason, intraperitoneal injection was chosen to administer AdipoRon in C26 mice.

C26 kinetic experiment

To study the kinetics of onset of cachexia, C26 animals were necropsied 8, 9, and 10 days after tumor cell injection in a separate experiment. These three time points correspond to different stages in the progression of cachexia in terms of body weight loss and food intake as already reported in a previous experiment [3]. Mice showed significant weight loss from day 9 (pre-cachexia) along with a significant depletion of muscle mass. Briefly, after one-week acclimatization, seven-week-old CD2F1 male mice (Charles River Laboratories, MA, USA) were injected subcutaneously in the upper flank with colon carcinoma C26 cells (1×10^6 cells/0.1 mL saline; C26 mice) or with saline solution (CT mice). Six groups of mice with equivalent body weight were formed: CT and C26 groups, each euthanized and necropsied 8, 9, and 10 days after tumor cell injection. Body weight was measured daily until euthanasia.

Grip strength test

Whole body grip strength was determined using a grip strength meter (Bioseb, Vitrolles, France). Mice were allowed to use their front and back paws to grab a grid and the tail was slowly pulled back by the same investigator. Tension was recorded at the time the mouse released the paws from the grid. Measurements were repeated three times, and the maximum tension of the three measurements was used in analyses. Each test was repeated twice.

Treadmill test

At the age of 15 weeks, mice were placed on a treadmill with an uphill inclination of 15° to assess their endurance and their resistance to fatigue. The initial speed was set at 5 m/min for 1 min, followed by a progressive increase in speed of 1 m/min up to 18 m/min. After that, the mice were allowed to run at 18 m/min until the end of the protocol (60 min in total). Fatigue was defined as the time at which mice were no longer able or willing to keep up with the treadmill despite gentle electric shock at the back of the treadmill.

Count of intestinal polyps in Apc$^{Min/+}$ mice

The small intestine was carefully excised and cut into three equal segments (upper, middle, and lower intestine). Large intestines were also dissected. All the segments were flushed with PBS, opened longitudinally, and flattened on paper towel. Total polyps in each section were counted by the same investigator and were categorized according to diameter size (<1 mm, 1-2 mm, 2-3 mm, 3-4mm and >4 mm).

mRNA analysis by RT-qPCR

Total RNA was extracted from frozen tissue samples using the TriPure isolation reagent (Roche Applied Science, Penzberg, Germany) as described by the manufacturer. cDNA was prepared by reverse transcription of 1 µg RNA using RevertAid H Minus Reverse Transcriptase, Random Hexamer, RiboLock RNase Inhibitor and dNTP Mix (all from Thermo Fisher Scientific, Waltham, MA, USA). Sybr Green® Real-time quantitative PCR (RT-qPCR) was performed as previously described [4]. Relative mRNA levels were calculated using the comparative CT method and normalized by the expression of housekeeping gene *Cyclophilin.* Primer sequences used for amplification during real-time qPCR are listed in Table S2 (Supplementary Materials). Primers were tested to avoid primer dimers, self-priming formation, or any unspecific amplification.

Mitochondrial DNA content analysis

Quadriceps tissue (20 to 30 mg) was digested with 0.1 mg/mL proteinase K (100 µg/mL) in lysis buffer (100mM Tris-HCL pH8, 1mM EDTA pH8, 100mM NaCl, 1% (w/v) SDS) at 50 °C overnight. Then, total DNA was isolated with phenol/chloroform/isoamyl alcohol method, pelleted with 100% ethanol and re-suspended in DEPC H_2O. To estimate mtDNA copy number, sybr Green® RT-qPCR was performed as previously described [4] using specific mitochondrial gene primers: Cytochrome B (*Cyt-b*) and NADH-ubiquinone oxidoreductase chain 1 (*Nd1*) and nuclear gene primers: H19 imprinted maternally expressed transcript (*H19*) listed in Table S2 (Supplementary Materials). Relative mitochondrial DNA content, corresponding to the ratio between mtDNA and nuclear DNA gene levels, was calculated using the comparative CT method and normalizing by the expression of *H19* gene.

Western blot analyses

Proteins of frozen skeletal muscle samples were homogenized in ice-cold buffer containing 20 mM Tris (pH 7), 270 mM sucrose, 5 mM EGTA, 1 mM EDTA, 1 mM sodium orthovanadate, 50 mM β-glycerophosphate, 5 mM sodium pyrophosphate, 50 mM sodium fluoride, 1 mM DTT, 1% v/v Triton X-100, and 10% protease inhibitor cocktail (Roche Applied Science). Homogenates were centrifuged at 10 000 g for 10 min at 4°C, and supernatants were immediately stored at −80°C. Total protein content was quantified using the Pierce BCA Protein Assay (Thermo Fisher Scientific). 30 µg of tissue proteins were resolved by sodium dodecyl sulfate-polyacrylamide gel 10% electrophoresis and transferred to PVDF

membranes. Membranes were incubated overnight at 4°C with the following primary antibodies diluted in 1% bovine serum albumin (BSA): anti-MuRF1 (1:150, AF5366, R&D Systems, Minneapolis, MN, USA), anti-K48-linkage specific polyubiquitin (1:1000, 8081, Cell Signaling Technology, Danvers, MA, USA), anti-phospho-Stat3 (1:500, 9145, Cell Signaling Technology) and anti-Stat3 (1:1000, 8768, Cell Signaling Technology) . Then membranes were incubated with a horseradish peroxidase (HRP) coupled to secondary antibody (Cell Signaling Technology) and revelation was done using Enhanced Chemiluminescence (ECL) Western blotting Detection System Plus (GE Healthcare, Chicago, IL, USA). The membranes were scanned with the Epson 60a and signal intensity was quantified using ImageJ software (NIH, http://imagej.nih.gov/ij). Signal intensity was normalized to whole lane of total protein loads assessed by Coomassie blue staining of the membrane.

Blood analyses

Blood samples were collected at sacrifice, allowed to clot for 30 min at room temperature and then centrifuged at 2000 g for 20 min at 4°C to obtain serum. Serum was aliquoted and stored at −80°C until analysis. Full-length adiponectin and IL-6 levels were measured with specific enzyme-linked immunosorbent assay (ELISA) kits (both from R&D Systems) according to the recommendations of the manufacturer. Total corticosterone was measured by competitive ELISA (MyBioSource, San Diego, USA) following the manufacturers' instructions. Concentrations of glycerol (Abcam, Cambridge, UK) and non-esterified fatty acids (NEFA; Randox, Crumlin, UK) were quantified by commercial kits based on colorimetric methods according to the manufacturer's instructions. Triglycerides levels were determined by colorimetric method FUJI DRI-CHEM NX500 biochemical analyzer (Fujifilm, Tokyo, Japan). For Apc mice, during tissue collection, a small part of blood was collected with heparinized capillary tubes, placed on ice, and centrifuged at 4000 rpm for 10 min using a centrifuge Haematokrit 210 (Hettich, Kirchlengern, Germany) to determine the haematocrit.

Muscle fiber cross-sectional area

Gastrocnemius muscle samples were fixed in 10% formalin for 48 h and embedded in paraffin. Five µm thick formalin fixed paraffin embedded (FFPE) tissue sections were mounted on SuperFrost Plus slides (Menzel-Gläser, Braunschweig, Germany), and deparaffinized with xylene and rehydrated in a graded series of ethanol baths. Gastrocnemius tissue sections were then stained with rhodamine-labeled Wheat Germ Agglutinin (WGA; Vector Laboratories

Newark, CA, USA) and Hoechst (Sigma, Darmstadt, Germany). Entire muscle sections were then scanned using the Axio Scan.Z1 slide scanner (Zeiss, Oberkochen, Germany) at 20X magnification. Five fields per section of each mouse (n=6 per group) were randomly pictured at magnification 20X using ZEISS ZEN 3.4 (blue edition) software (Zeiss). Cross-sectional area was determined for each fiber using ImageJ 1.47v software (NIH, Bethesda, MD, USA).

Adipocyte cross-sectional area

Epipidymal white adipose tissue samples were fixed in 10% formalin for 48 h and then embedded in paraffin. Five µm thick FFPE tissue sections were mounted on SuperFrost Plus slides (Menzel-Gläser), and deparaffinized with xylene and rehydrated in a graded series of ethanol baths. Adipose tissue sections were then stained with mayer haematoxylin (Sigma) and Erythrosine B (Merck, Darmstadt, Germany) and after scanned under Pannoramic Scan II slide scanner (3DHISTECH, Budapest, Hungary) at 20X magnification. Five fields per section were randomly pictured using slideviewer software (3DHISTECH). Adipocyte diameter was measured automatically using Adiposoft 1.16 software (under ImageJ 1.47win software) followed by manual correction.

Clinical study in cancer patients

Patients with colorectal or lung cancer, confirmed by pathology, were enrolled in a cross-sectional prospective study (NCT01604642) at the time of diagnosis or at relapse and before any therapeutic intervention, between January 2012 and March 2014 at the Cliniques universitaires Saint-Luc, Brussels, Belgium. The study protocol was approved by the local ethics committee of the UCLouvain (B403201111269) and written consent was given by patients prior to entry into the study. Exclusion criteria were: non-caucasian subjects, obvious malabsorption, major depression, artificial nutrition, high doses of steroids (>1 mg/kg hydrocortisone equivalent), hyperthyroidism, other causes of malnutrition, major walking handicap, ECOG performance status ≥4 and psychological, familial, social or geographic conditions that would preclude participation in the full protocol. The diagnosis of cachexia was established according to the definition proposed by Fearon et al. [5], as at least one of these criteria: an involuntary weight loss > 5% over the past 6 months; or weight loss > 2% and body mass index (BMI) < 20 kg/m^2; or weight loss > 2% and low muscularity. Patient distribution is reported in supplementary materials (Table S3). Blood samples were collected from cancer patients at the time of recruitment, in standardized conditions. Plasma was aliquoted and stored

at −80°C until analysis. Plasma sample from age-matched healthy subjects was used as control group (CT). Plasma human total adiponectin levels was measured with ELISA kit (R&D Systems) according to the recommendations of the manufacturer.

SUPPLEMENTARY TABLES

Table S1. Criteria for murine sepsis score determination

Murine Sepsis Score				
**Score**	**0**	**1**	**2**	**3**
**Appearance**	Smooth coat	Slightly ruffled fur	Majority of fur on back is ruffled	Piloerection, puffy appearance
**Level of consciousness**	Active	Active, avoids standing upright	Active only when provoked	Non-responsive, even when provoked
**Activity**	Normal	Suppressed eating, drinking, or running	Stationary	Stationary, even when provoked
**Response to stimulus**	Normal	Slowed response to auditory or touch stimuli	No response to auditory, slowed response to touch	No response to touch stimuli
**Eyes**	Open	Not fully open, potentially secretions	Half closed, potential secretions	Mostly or completely closed
**Respiration quality**	Normal	Periods of labored breathing	Consistently labored breathing	Labored breathing with gasps

Murine Sepsis Score scoring system for evaluating disease severity in C26 mouse model, adapted from [6].

Table S2. List of primer sequences used for real-time quantitative PCR analysis

Gene name	Target	Forward (5′-3′)	Reverse (5′-3′)
Bnip3	cDNA	CCCGCTGGCCTCCAACA	GGAAGGAACGTGCTCCAG
Cd36	cDNA	GGCCAAGCTATTGCGACAT	CAGATCCGAACACAGCGTAGA
Cd68	cDNA	CTTCCCACAGGCAGCACAG	AATGATGAGAGGCAGCAAGAGG
Cd163	cDNA	TGCTGTCACTAACGCTCCTG	TCATTCATGCTCCAGCCGTT
Complément C3	cDNA	ACTGAAGGTCGTGCCAGAAG	GGGGTCACGATCAGGTGTTT
Cpt1a	cDNA	CTGAGCCATGAAGCCCTCAA	CACACCCACCACCACGATAA
Cyclophilin D	cDNA	TAAGCATGATCGGGAGGGTT	CGTCCAGATGAGGAGTCGGA
Ddit4	cDNA	AGACTCCTCATACCTGGATGGG	AGCTGCATCAGGTTGGCAC
Fasn	cDNA	AAGCGGTCTGGAAAGCTGAA	GAAGCGTCTCGGGATCTCTG
Fbxo32 (Atrogin1)	cDNA	CCATCAGGAGAAGTGGATCTATGTT	GCTTCCCCCAAAGTGCAGTA
Foxo1	cDNA	TCAAGGATAAGGGCGACAGC	GTTCCTTCATTCTGCACTCGAAT
Foxo3	cDNA	CGGCTCACTTTGTCCCAGA	GCCGGATGGAGTTCTTCCA
Gabarapl1	cDNA	GAGGACCACCCCTTCGAATATC	CAGTGAGGTCGGAGGGCA
Gpat1	cDNA	GTTGAACTCCTCCGACAGCA	GCTCTCGTTCTTCTTGGGCT
Gpihbp1	cDNA	AGGGCTGTCCTCCTGATCTT	AGAAGGGTTTGCTGCTGGAG
Hp	cDNA	TATCGCTGCCGACAGTTCTAC	CGTGGCGGGAGATCATCTTG
IkBa	cDNA	TGGCCTTCCTCAACTTCCAG	GCCAGCTTTCAGAAGTGCCT
Il6	cDNA	CCAGAGATACAAAGAAATGATGG	ACTCCAGAAGACCAGAGGAAA
Il1b	cDNA	TTTGAAGTTGACGGACCCCA	CCTCATCCTGGAAGGTCCAC
Lipe	cDNA	TGTCACGCTACACAAAGGCT	GGTCACACTGAGGCCTGTC
Lpl	cDNA	ATCGGGCCCAGCAACATTAT	CTGGGGGCTTCTGCATACTC
Map1lc3b	cDNA	ATGCCGTCCGAGAAGACCTT	ATCACTGGGATCTTGGTGGG
Mef2c	cDNA	GCTGTTCCAGTACGCCAGCAC	AGTGCGTGGGGTGAGTGCATAA
Mrf4	cDNA	CCCAAGGTGGAGATTCTGCG	CTGAGGCATCCACGTTTGCT
Murf1	cDNA	TGTCTGGAGGTCGTTTCCG	ATGCCGGTCCATGATCACTT
Myh1	cDNA	AGCTTCAAGTTTGGACCCACGGTCG	GCAGCCTCCCCGAAAACGGC
Myh2	cDNA	GCCTAAGGTGGTCAAAGGCA	GGCAGCTCCACCACTACTTG
Myh4	cDNA	CGCATCTGTAGGAAGGGGTT	TCT-AGA-GTT-CCC-AAC-AGG-CCG
Myh7	cDNA	GGTGCCAAGGGCCTGAATGAGGAG	GGTCTGAGGGCTTCACGGGCAC
MyoD	cDNA	TCCGTGTTTCGACTCACCAG	AGAAGTGTGCGTGCTCTTCC
NFkb2	cDNA	CCTTCGTAGTTACAAGCTGGC	GGCACTGTCTTCTTTCACCT
Nik	cDNA	CGAGCTACTTCAACGGGGTC	GGCAATGTCTCCCACCTTGA
Osm	cDNA	CCAGTATGCAGACACGGCTT	CGTGAGGTTCGCCTGATTCT
PnPla2	cDNA	TTCGCAATCTCTACCGCCTC	AGCAAAGGGTTGGGTTGGTT
Relb	cDNA	TTCAAAACGCCACCCTACGA	ACACCGTAGCTGTCATGATCC
Serpina3n	cDNA	GACCTGTCTGCAATCACAGGA	TTTGGGGTTGGCTATCTTGGC
Rn18S RNA	cDNA	GCAATTATTCCCCATGAACG	GGCCTCACTAAACCATCCAA
Rn28S RNA	cDNA	AAGCGTTGGATTGTTCACCC	TCCTCAGCCAAGCACATACA
Rn45S pre-rRNA (ITS)	cDNA	CCGGCTTGCCCGATTT	GCCAGCAGGAACGAAACG

Socs3	cDNA	GCCACTTCTTCACGTTGAGC	GTCACTCTGCAGCGAAAAGC
Tnfa	cDNA	GATCGGTCCCCAAAGGGATG	TGGTTTGCTACGACGTGGG
Ucp1	cDNA	CGTCCCCTGCCATTTACTGT	GACCCGAGTCGCAGAAAAGA
Vegf	cDNA	GAGCGTTCACTGTGAGCCTTG	TGCAACGCGAGTCTGTGTTT
Cytochrome B	mtDNA	ATTCCTTCATGTCGGACGAG	ACTGAGAAGCCCCCTCAAAT
H19	nuclear DNA	GTACCCACCTGTCGTCC	GTCCACGAGACCAATGACTG
Nd1	mtDNA	AATCGCCATAGCCTTCCTAACAT	GGCGTCTGCAAATGGTTGTAA

Table S3. Characteristics of healthy subjects and cancer patients

	Healthy subjects	Colorectal cancer patients		Lung cancer patients	
	**Control**	**Non-cachectic**	**Cachectic**	**Non-cachectic**	**Cachectic**
**n**	14	31	23	17	15
**Age (years)**	67.1 (61–77)	64.0 (25–83)	67.1 (37–88)	69.1 (43–86.0)	69.2 (49-84)
**BMI (kg/m^2)**	24.9 (21.2-31.2)	27.6 (15.6-45.4)	26.5 (21.3-35.8)	28.7 (23.5–34.8)	23.5 (17.7–27.5)
**Weight loss (%)**	0	0.8 (0–3.7)	8.2 (2–25)	1.2 (0–4)	9.1 (2.5-21.1)

BMI: body mass index.

SUPPLEMENTARY FIGURES

Fig. S1

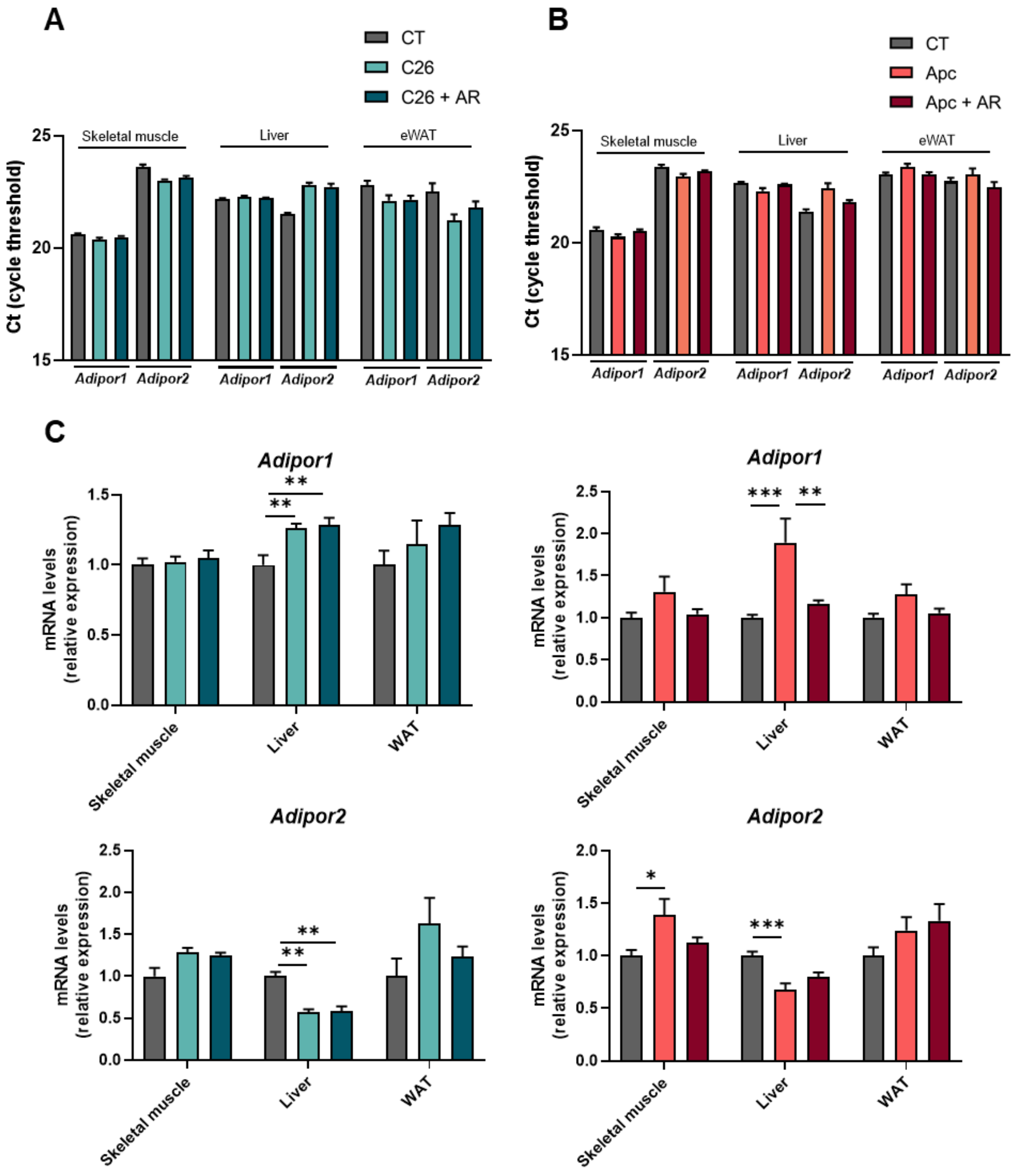

Figure S1. Effects of AdipoRon on Adiponectin receptor (*AdipoR1* and *AdipoR2*) gene expression in cachectic C26 and Apc$^{Min/+}$ mice.

(A) Comparison of AdipoR gene (*Adipor*1 and *Adipor2*) cycle thresholds (Ct) from RT-qPCR analysis in skeletal muscle, liver and fat (eWAT) of sham-injected mice (CT), untreated C26 mice (C26) and AdipoRon-injected C26 mice (C26+AR) 11 days after cancer cells injection (n = 7-8/group). The lowest Ct indicates the highest expression of *AdipoR* among tissues analyzed.

(B) Comparison of AdipoR gene (*Adipor*1 and *Adipor2)* cycle thresholds (Ct) from RT-qPCR analysis in skeletal muscle, liver and fat (eWAT) of C57BL/6J wild-type mice (WT), Apc$^{Min/+}$ mice (Apc), and Apc$^{Min/+}$ mice receiving AdipoRon in their water (Apc+AR) at 20 weeks (n = 9-12/group). (C) Relative gene expression levels of *Adipor1* and *Adipor2* in skeletal muscle, liver and fat in C26 mice (n = 7-8/group) and in Apc$^{Min/+}$ mice (n = 15-16/group). Data are reported as mean ± SEM. Significant differences are indicated as *p < 0.05, **p < 0.01 and ***p < 0.001.

Fig. S2

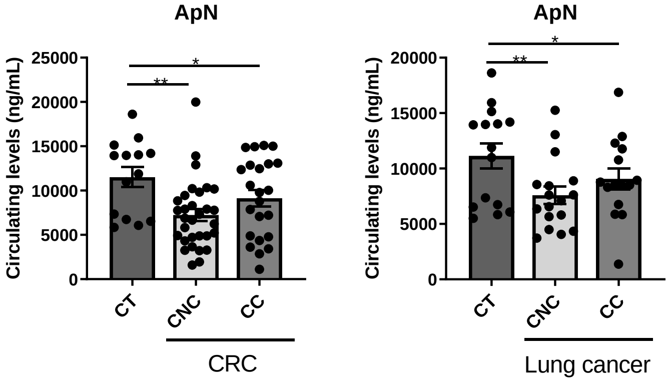

Figure S2. Adiponectin levels are reduced during cancer.

Total adiponectin (ApN) levels in plasma of healthy subjects (CT), non-cachectic (CNC) and cachectic (CC) cancer patients with either colorectal cancer (CRC) (CT, n = 14; CNC, n = 31; CC, n = 23) or lung cancer (CT, n = 14; CNC, n = 17; CC, n = 15). Data are reported as mean ± SEM. Significant differences are indicated as *p < 0.05 and **p < 0.01.

Fig. S3

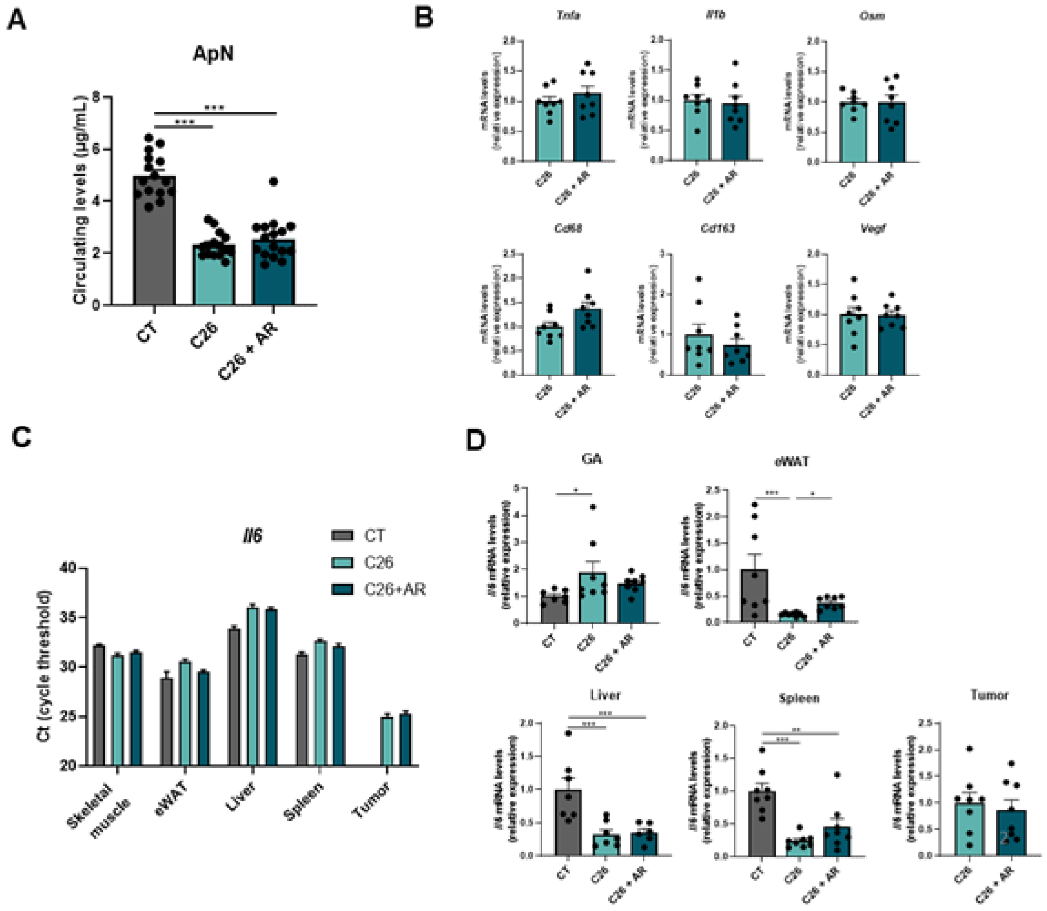

Figure S3. Effects of AdipoRon on adiponectin levels, tumoral gene expression and tissue IL-6 expression in cachectic C26 mice.

(A) Serum adiponectin (ApN) levels of sham-injected mice (CT), untreated C26 mice (C26) and AdipoRon-injected C26 mice (C26+AR) 11 days after cancer cells injection (n = 15-16/group). (B) Relative expression mRNA levels of main pro-inflammatory cytokines, markers of macrophages, Vascular Endothelial Growth Factor (*Vegf*) and Oncostatin M (*Osm*) in tumor of untreated mice (C26) and AR-treated C26 mice (C26 +AR) at day 11. (C) Comparison of *Il6* cycle thresholds (Ct) from RT-qPCR analysis between groups for gastrocnemius muscle (GA), fat (eWAT), liver, spleen and tumor of sham-injected mice (CT), C26 mice (C26) and AR-treated C26 mice (C26+AR) at day 11. The lowest Ct indicates the highest expression of *Il6* among tissues analyzed. (D) Relative mRNA expression levels of *Il6* in GA, fat, liver and polyps of mice from the three groups at day 11. Data are reported as mean ± SEM (n = 8-16/group). Significant differences are indicated as *p < 0.05, **p < 0.01 and ***p < 0.001.

Fig. S4

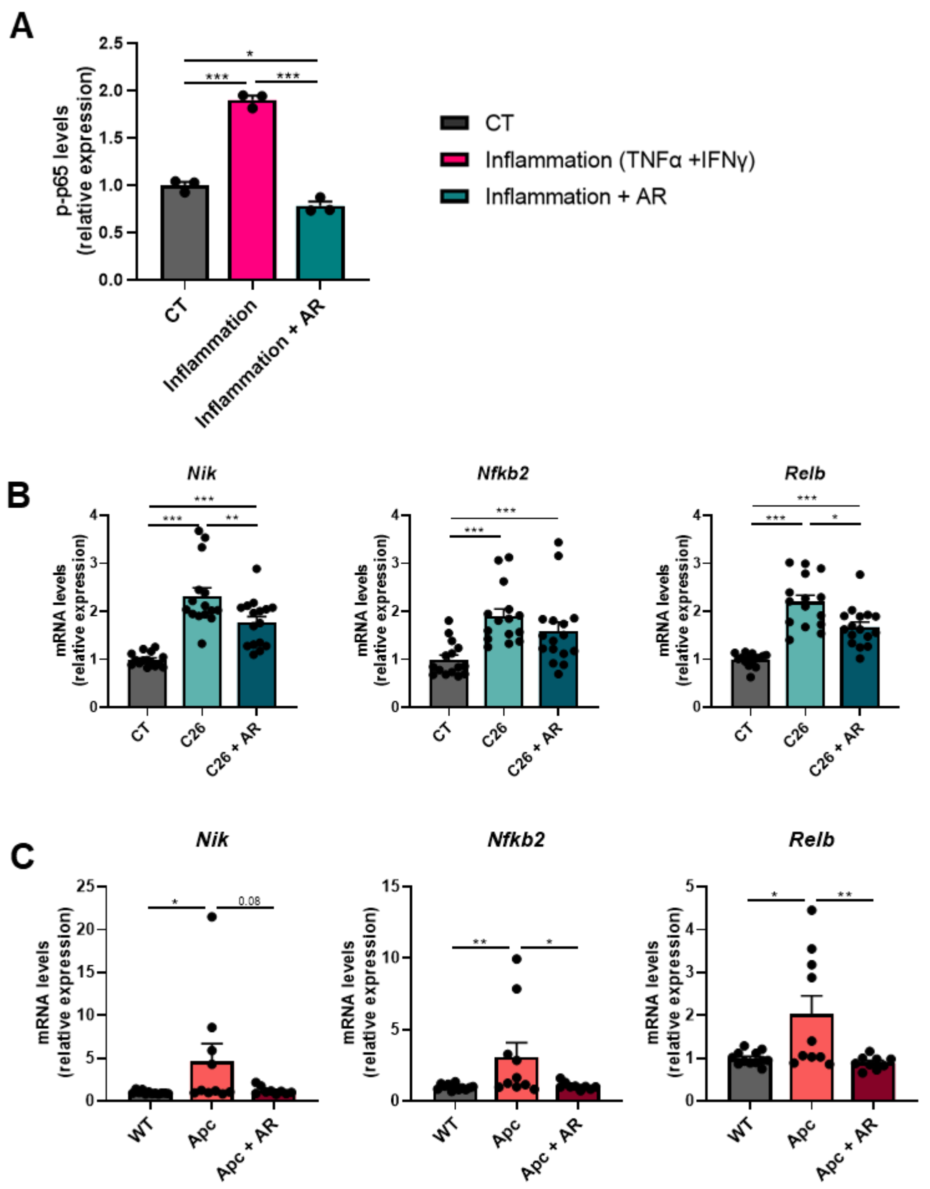

Figure S4. Effects of AdipoRon on canonical and non-canonical NF-KB pathways in cachectic C26 and Apc$^{Min/+}$ mice.

(A) Effects of AdipoRon on p65 phosphorylation in C2C12 myotubes challenged by pro-inflammatory cytokines. Cells were treated with or without AdipoRon (25 µM), while being challenged or not with TNFα (10 ng/mL) and IFNγ (10 ng/mL) for 24 h (n=3/group; three independent experiments). (B) Relative gene expression levels of *Nik, Nfkb2* and *Relb* in skeletal muscle of C26 mice (n = 15-16/group). (C) Relative gene expression levels of *Nik, Nfkb2* and *Relb* in skeletal muscle of Apc$^{Min/+}$ mice (n = 9-12/group). Data are reported as mean ± SEM. Significant differences are indicated as *p < 0.05, **p < 0.01 and ***p < 0.001.

Fig. S5

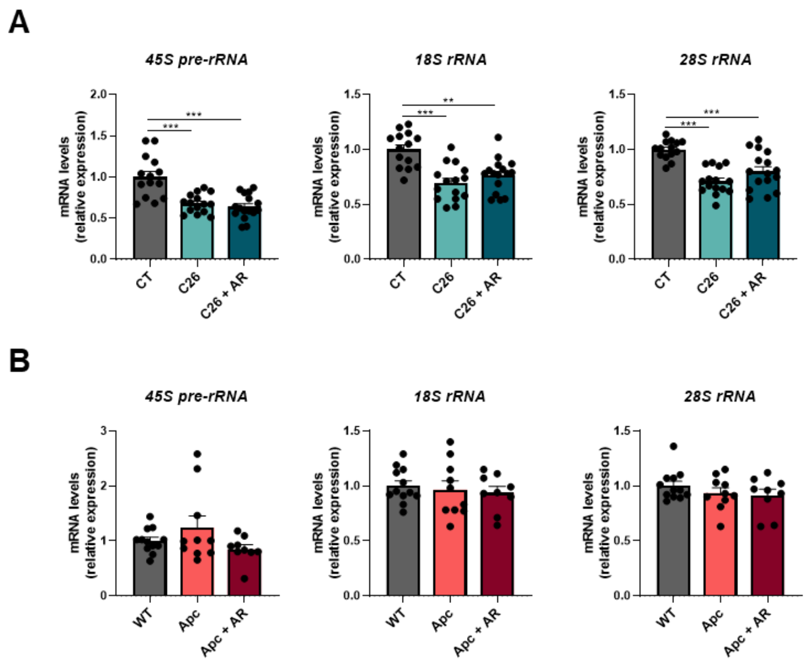

Figure S5. Effects of AdipoRon on ribosomal RNA content in skeletal muscle of cachectic C26 and Apc$^{Min/+}$ mice.

Relative 45S pre-rRNA (ITS; Internal Transcribed Spacer), 18S and 28s rRNA content (ribosomal subunit 40S and 60S respectively) in gastrocnemius muscle of CT, C26 and C26+AR mice (A; n = 14-16/group) and of WT, Apc$^{Min/+}$ and Apc$^{Min/+}$+AR mice (B; n = 9-12/group). Data are reported as mean ± SEM. Significant differences are indicated as **p < 0.01 and ***p < 0.001.

Fig. S6

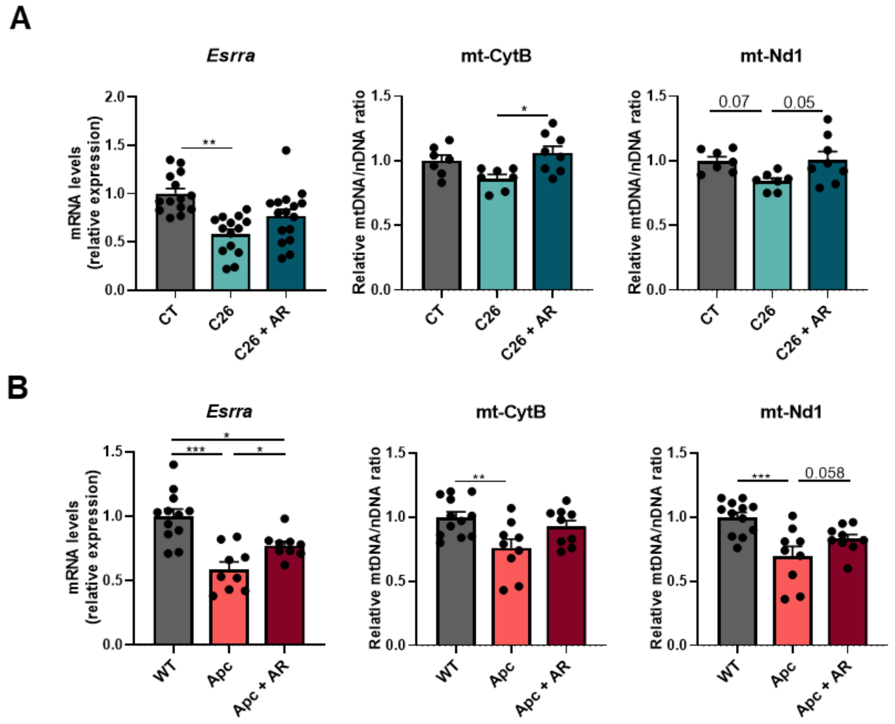

Figure S6. Effects of AdipoRon on gene expression of *Esrra*, a transcriptional regulator of mitochondrial biogenesis, and on mitochondrial content in skeletal muscle of cachectic C26 and Apc$^{Min/+}$ mice.

Relative gene expression of ERRα (*Esrra*) and mitochondrial DNA (mtDNA) content, determined by ratio between mitochondrial (*CytB* and *Nd1*) and nuclear (*H19*) DNA genes, in quadriceps muscle of CT, C26 and C26+AR mice (A; n = 7-16/group) and of WT, Apc$^{Min/+}$ and Apc$^{Min/+}$+AR mice (B; n = 9-12/group). Data are reported as mean ± SEM. Significant differences are indicated as *p < 0.05, **p < 0.01 and ***p < 0.001.

Fig. S7

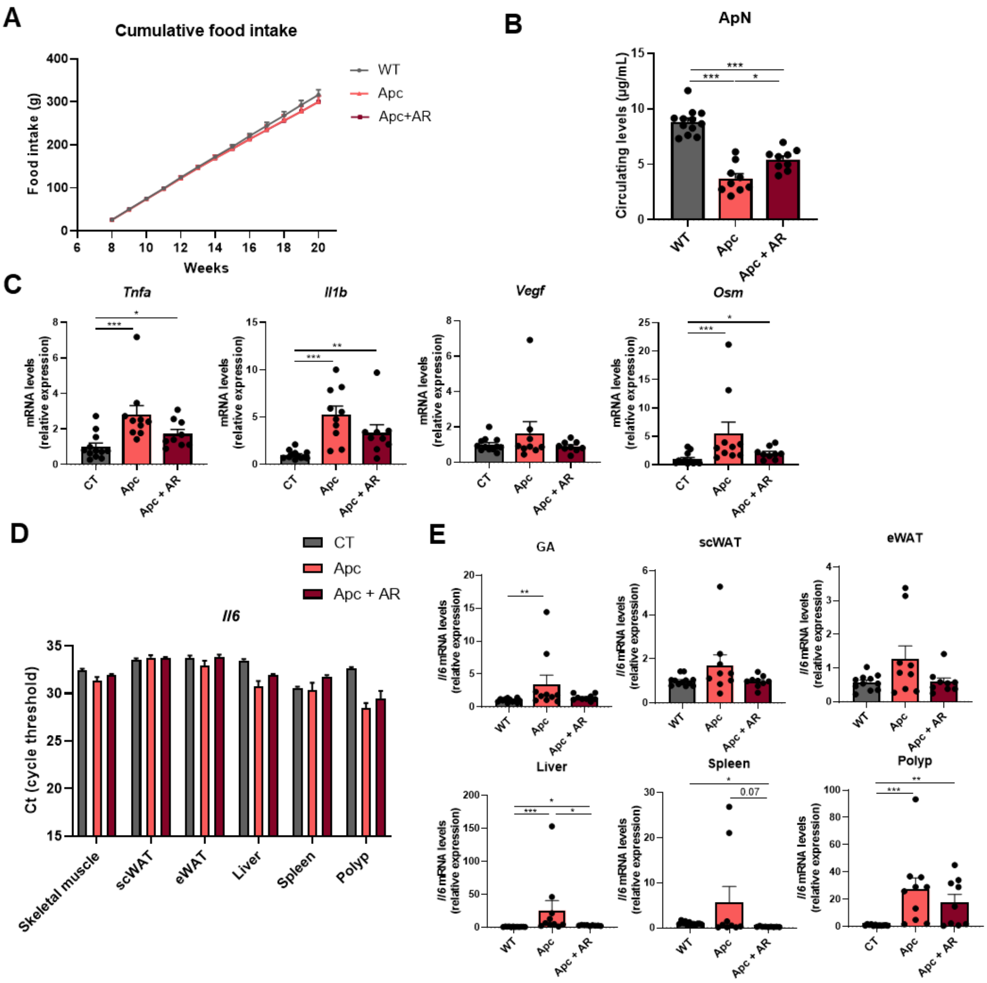

Figure S7. Effects of AdipoRon on food intake, adiponectin levels, polyp gene expression and tissue IL-6 expression in cachectic Apc$^{Min/+}$ mice.

(A) Cumulative food intake over a 12-week period in C57BL/6J wild-type mice (WT), Apc$^{Min/+}$ mice (Apc) and in AR-treated Apc$^{Min/+}$ mice (Apc + AR). (B) Serum adiponectin (ApN) at 20 weeks. (C) Relative expression mRNA levels of pro-inflammatory cytokines (*Tnfa*, *Il1b*), Vascular Endothelial Growth Factor (*Vegf*) and Oncostatin M (*Osm*) in intestinal tract of WT mice and in polyps of mice from the two Apc groups at 20 weeks. (D) Comparison of *Il6* cycle thresholds (Ct) from RT-qPCR analysis between the three groups for gastrocnemius muscle (GA), fat (scWAT and eWAT), liver, spleen and polyps. The lowest Ct indicates the highest expression of *Il6* among tissues analyzed. (E) Relative mRNA expression levels of *Il6* in GA, fat, liver, spleen and polyps of mice from the three groups. Data are reported as mean ± SEM (n = 9-12/group). Significant differences are indicated as *p < 0.05, **p < 0.01 and ***p < 0.001.

SUPPLEMENTARY REFERENCES

1. Sciorati, C., et al., *Necdin is expressed in cachectic skeletal muscle to protect fibers from tumor-induced wasting.* J Cell Sci, 2009. **122**(Pt 8): p. 1119-25.
2. Okada-Iwabu, M., et al., *A small-molecule AdipoR agonist for type 2 diabetes and short life in obesity.* Nature, 2013. **503**(7477): p. 493-499.
3. Thibaut, M.M., et al., *Inflammation-induced cholestasis in cancer cachexia.* J Cachexia Sarcopenia Muscle, 2021. **12**(1): p. 70-90.
4. Massart, I.S., et al., *Marked Increased Production of Acute Phase Reactants by Skeletal Muscle during Cancer Cachexia.* Cancers, 2020. **12**(11): p. 3221.
5. Fearon, K., et al., *Definition and classification of cancer cachexia: an international consensus.* Lancet Oncol, 2011. **12**(5): p. 489-95.
6. Mai, S.H.C., et al., *Body temperature and mouse scoring systems as surrogate markers of death in cecal ligation and puncture sepsis.* Intensive Care Med Exp, 2018. **6**(1): p. 20.
